# Pooling and Analysis of Published *in Vitro* Data: A Proof of Concept Study for the Grouping of Nanoparticles

**DOI:** 10.3390/ijms161125954

**Published:** 2015-11-02

**Authors:** Myrtill Simkó, Sonja Tischler, Mats-Olof Mattsson

**Affiliations:** Health & Environment Department, AIT Austrian Institute of Technology GmbH, Konrad-Lorenz-Straße 24, Tulln 3430, Austria; sonja@tischler.io (S.T.); Mats-Olof.Mattsson@ait.ac.at (M.-O.M.)

**Keywords:** nanoparticles, toxicity, intracellular ROS, cell viability, phagocytic and non-phagocytic cells, CNT

## Abstract

The study aim was to test the applicability of pooling of nanomaterials-induced *in vitro* data for identifying the toxic capacity of specific (SiO_2_, TiO_2_, ZnO, CuO, CeO_2_ and carbon nanotubes, [CNT]) nanoparticles (NP) and to test the usefulness for grouping purposes. Publication selection was based on specific criteria regarding experimental conditions. Two relevant biological endpoints were selected; generation of intracellular reactive oxygen species (ROS) and viability above 90%. The correlations of the ROS ratios with the NP parameters’ size, concentration, and exposure time were analysed. The obtained data sets were then analysed with multiple regression analysis of variance (ANOVA) and the Tukey post-hoc test. The results show that this method is applicable for the selected metal oxide NP, but might need reconsideration and a larger data set for CNT. Several statistically significant correlations and results were obtained, thus validating the method. Furthermore, the relevance of the combination of ROS release with a cell viability test was shown. The data also show that it is advisable to compare ROS production of professional phagocytic with non-phagocytic cells. In conclusion, this is the first systematic analysis showing that pooling of available data into groups is a useful method for evaluation of data regarding NP induced toxicity *in vitro*.

## 1. Introduction

According to Krug [1], the number of published studies regarding the hazards of engineered nanoparticles (NP) increased almost exponentially since the year 2000 to more than a total of 10,000 publications in 2014. Despite this staggering amount of publications, there are still substantial knowledge gaps that impede a proper risk assessment of NP. Such knowledge gaps cover many areas, including NP characterization, exposure assessment, and hazard identification and characterization.

There are not only a large amount of published studies regarding the safety of nanomaterials, there are also an abundance of nanomaterials and modifications of these, regarding size, shape, surface properties, *etc.* It is also expected that novel materials will appear at least as frequently in the future as in the recent past. Naturally, this raises the question of how to perform safety assessments for so many (different) materials. Is it necessary to perform assessments of each individual material and its variations, or is it feasible to adopt a category approach? The latter alternative, also called “grouping”, is established for chemicals, where testing is performed on certain substances, and the results are considered applicable for other closely related compounds (The Organisation for Economic Co-operation and Development, OECD 2014 [2]).

A need for grouping of nanomaterials has been voiced by many authors (e.g., [3,4], among others), although no consensus agreement on any specific grouping approach has been reached. Naturally, any grouping effort cannot be better than the data that underpin the activity, so a primary requisite for grouping must be that available scientific studies are of high quality and provide reliable and useful data. It is from this perspective that the present study should be seen, namely as a pilot effort to evaluate the usefulness of specific toxicity-related data regarding selected NP.

A number of projects have been initiated to e.g., develop and refine NP-testing approaches, such as the EU-funded NanoSafety Cluster [5] and the ITS-NANO project [6]. Other projects deal with answering regulation-related questions from the national regulation and legislation authorities but also from the society and the industry (www.nanoreg.eu). OECD is running a large programme addressing the need for comprehensive NP risk assessments for human health and the environment (www.oecd.org/science/nanosafety). However, while the applicability of a number of testing strategies is discussed, the question remains whether the already available studies regarding particular NP toxicity or specific mode of action mechanisms can be used for assessment of NP toxicity. Thus, the question is whether it is possible to extract relevant toxicity related information from a selected number of studies leading to answers about the toxicity of a specific NP?

One of the key characteristics of an NP is the increased surface to volume ratio compared to larger particles. The surface area of a nanoparticle (NP) is more reactive than that of the bulk material due to physical and chemical properties. Accordingly, the behaviour of an NP is expected to change with changing size, which also has been documented in many studies [7,8,9,10,11].

Furthermore, the NP size can determine the pathway of cell entry [12,13]. NPs are not restricted to one but can activate multiple pathways to enter the cell. These uptake routes include phagocytosis (mainly used by professional phagocytic cells such as monocytes, neutrophils or macrophages) [14]; clathrin-dependent endocytosis which is reported as the main pathway for NP uptake and has been documented in e.g., muscle cells, adipocytes and endothelial cells [11]; clathrin- and caveolin-independent endocytosis (less frequently employed by NP [11], but plays a role in signal transduction and transcytosis); and macropinocytosis which is considered as a non-specific entry-point detected in various cell types (e.g., [14]). Iversen *et al.* [15] summarized that since the size of caveolae are around 50–80 nm, the uptake and accumulation of larger NP is very unlikely. Mao *et al.* [16] demonstrated that clathrin-mediated endocytosis is “limited” by around 120 nm, caveolin-mediated by *ca*. 60 nm, and clathrin/caveolin independent endocytosis by around 90 nm, whereas phagocytic uptake can process objects in the μm range. Similar findings have been shown by Jana [12] as well. Shang *et al.* [17] summarised the influence of size-dependent NP uptake: “(i) There is an optimal size for efficient endocytosis of NP independent of the particle composition; (ii) This critical size can vary with cell type and surface properties of the NP; (iii) Small NP have a higher probability to be internalized by passive uptake than large ones; (iv) Under otherwise identical conditions, small NP are more likely to cause toxic cellular responses…”

The NP size related influence on cells is usually related to the primary size of the particle which depends on the NP synthesis method [18]. Which pathway or entry-point is activated by the NP uptake is furthermore influenced by the particles agglomeration, aggregation and/or stability. In the present study, the mean primary NP sizes are considered.

Endocytic uptake takes only a few hours *in vitro* (e.g., [19]). After 2 h, the percentage of internalized particles reaches a plateau phase and the cell is more or less full with particles, which is a non-physiologic condition *in vitro*. Still, many *in vitro* studies report about the effects after longer durations, up to 24 h. Such studies can generate false positive results or simply wrong effects since during this time period and condition, other processes can be induced, such as apoptosis, necrosis, or changes in cell proliferation.

Another relevant parameter is the shape of the NP which might play an important role in cellular uptake and toxicity, as demonstrated by Gratton *et al.* [19]. The authors detected differences in the internalization of cubical and cylindrical NPs with various aspect ratios. Furthermore, the authors described that rod-like particles were favoured by the investigated cells. These findings were confirmed by Oh and Park [20]. It was concluded that macrophages are more efficient in rod-shaped NP uptake, while cancer and lung epithelial cells internalize spherical NP more efficiently. The fibrous forms of nanotubes can lead to frustrated phagocytosis and increased toxicity if they are enclosed too long by the cell [21].

In many publications, the applied NP concentrations range between very low (<10 μg/mL) and very high levels (>100 μg/mL). In general, concentrations above 50 μg/mL are considered excessive and not relevant for toxicological studies (e.g., [22]). Furthermore, high NP concentrations *in vitro* do not correlate with *in vivo* test concentrations, thus reducing the significance of the experiment [22]. The effects of extremely low concentrations (pg/mL) are hardly ever analysed, but can alter cell signalling and cause sustained stress without being cytotoxic as it was shown in a chronic exposure *in vitro* study [23]. Most *in vitro* experiments are designed as acute toxicity tests or as mechanistic studies, as only few chronic *in vitro* assays have been developed so far.

There are other parameters that influence the NP-cell interactions. A listing of these would be beyond the scope of this study, but a comprehensive overview of such parameters is given by Nel *et al.* (e.g., [24]). The role of the cell culture medium should be mentioned, however, since it influences NP characteristics such as the zeta-potential, dispersion, and aggregation due to its pH, ionic strength, temperature, or presence of organic molecules or proteins.

In summary, the many publications concerning the toxicity of nanomaterials has led to only a limited overview or understanding of possible adverse effects, since it is not clear how to weigh the results of the published data. Our hypothesis is that the present approach allows the pooling of available nanotoxicity related data into more simplified categories of experimental conditions without changing the outcome, presenting a comparative overview of the available data, and identifying the toxic capacity of a specific NP. Thus, in the present study, the aim was (1) to identify specific and relevant experimental parameters for the chosen six different NP; and (2) to classify and implement “groups”, leading to a better understanding of biological effects of nanomaterials.

To perform this proof of concept study, appropriate biological endpoints were identified, serving as the basis for the comparative analysis. These endpoints guided the selection of which specific nanoparticles (SiO_2_, TiO_2_, ZnO, CuO, CeO_2_/Ce_2_O_3_) to investigate and the identification of the relevant experimental parameters (size, concentration, and exposure time). Additional parameters such as cell type, positive and negative control *etc.* were taken into account to analyse the quality of the published data set.

## 2. Results and Discussion

### 2.1. Intracellular ROS Release and Cell Viability as a Group of Biological Endpoints

The first parameter to identify was a relevant and frequently used biological endpoint, investigated with similar and, thus, comparable techniques. Thus, the release of reactive oxygen species was chosen. ROS release often leads to oxidative stress, which is considered as an early event of NP-induced adverse effects, but not necessarily leading to cell death, and is, therefore, an important toxicological parameter. Intracellular ROS release is commonly analysed with a specific technique, using the dichloro-dihydro-fluorescein diacetate (DCFH-DA) fluorescent probe, which facilitates data comparison.

While collecting the “all data” (see Material and Methods), the results of the cell viability tests were noted during the experiments, and, thus, under the same experimental conditions as the measurement of ROS release, a cell viability decrease was consistently shown. There are different explanations for this effect, however, during the cell death process (apoptosis or necrosis), the cell membrane disintegrates and so the fluorescent dye dichloro-dihydro-fluorescein (DCF) can leak out. It can be assumed that the measured intensity of DCF is not solely caused by intracellular ROS generation, but also by other unknown effects and, hence, easily leads to misinterpretations. Therefore, cell viability was considered as a second relevant biological endpoint when data were collected. Consequently, after eliminating all data points where the cell viability was given and was below 90%, the remaining data set was the second group of interest. In other words, the data set “>90% viability” contains data points with cell viability above 90%. Unfortunately, not all publications have included a cell viability test. However, the cell metabolic activity assay (MTT) was by far the most commonly used test, and in case more than one cell viability tests was employed, the results of the MTT assay were used. In case of CNT data, different cell viability tests were employed, since the tetrazolium salts reacts with the carbon nanotubes leading to false conclusions [25].

### 2.2. Analysis of the Selected Data

Initially, 102 publications were selected, whereupon a more or less equal number of publications were considered for each NP. The main focus was to perform a proof of concept study testing the applicability of the “pooling approach” rather than to analyse all available studies. From the selected publications, 36 were excluded because these did not fulfil the selection criteria (see Material and Methods). Of the remaining 66 publications, 11 examined more than one type of NP.

As one quality criterion, the physicochemical characterization of the NP was considered. Thus, nine studies did not present a complete description of the employed NP and referred to the supplier data only. Also, only about one third of all publications specified surface area and zeta potential, respectively. However, several authors gave detailed descriptions of the NP including impurities and behaviour of the NP in cell culture media and water.

In the studies, 43 different cell types were used, of which eight can be classified as professional phagocytes. Three different primary cells were used in the selected publications. About half of the cell types were of human origin, a quarter murine and the rest were derived from rats and fish. Information about the cells in general, such as age and storage or number of passages, was generally sparse, while descriptions of culture conditions during the experiments and parameters were given routinely.

Out of the considered 66 publications, 12 contained data points with cell viability below 90%. In total, 616 data points were read out from all publications of which 67% showed cell viability above 90%. Table 1 gives an overview of the number of investigations gathered from the selected publications. (A complete list of the publications is provided in the Table S1.)

**Table 1:** Number of investigations according to each NP in total and the >90% viability data.

NP	All Data	>90% Viability	Percentage of >90% Viability to All Data
CeO_2_	109	102	94%
CuO	40	28	70%
ZnO	166	82	49%
TiO_2_	75	49	65%
SiO_2_	108	73	68%
CNT	113	75	66%
Sum of investigations	611	409	67%

### 2.3. The Pooling Approach—Choosing Groups

Here, we selected the NP size, concentration, and the exposure time as relevant experimental conditions for the pooling. It has to be pointed out, that all extracted data represent the published ROS ratios that were here transformed to a comparable measure. Thus, the analysis is restricted to relative comparisons. We aimed to present *in vitro* data regarding ROS production and cell survival rate after exposure to the chosen NP with very low, low, medium, high, and very high/large/long NP sizes, concentrations and exposure times, respectively, since these characteristics/conditions are expected to be important for NP effects. Thus, group-ranges (groups) were chosen as presented in Table 2.

**Table 2:** Classification of data points into NP-size groups, NP-concentration groups and exposure time groups. If possible, each group contains at least five data points.

NP Size Groups (without CNT)	NP Size Groups (CNT)	NP Concentration Groups	Exposure Time Groups
≤15 nm	≤5 nm thick *x* ≤ 10 µm long	≤10 µg/mL	≤3 h
16–30 nm	>5–10 nm thick *x* ≤ 10 µm long	11–30 µg/mL	>3–12 h
31–50 nm	>10–50 nm thick *x* ≤ 10 µm long	31–50 µg/mL	>12–24 h
51–100 nm	>50 nm thick *x* ≤ 10 µm long	51–100 µg/mL	>24 h
	≤5 nm thick *x* > 10 µm long	>100 µg/mL	
****	>5–50 nm thick *x* > 10 µm long		

**Figure 1:**
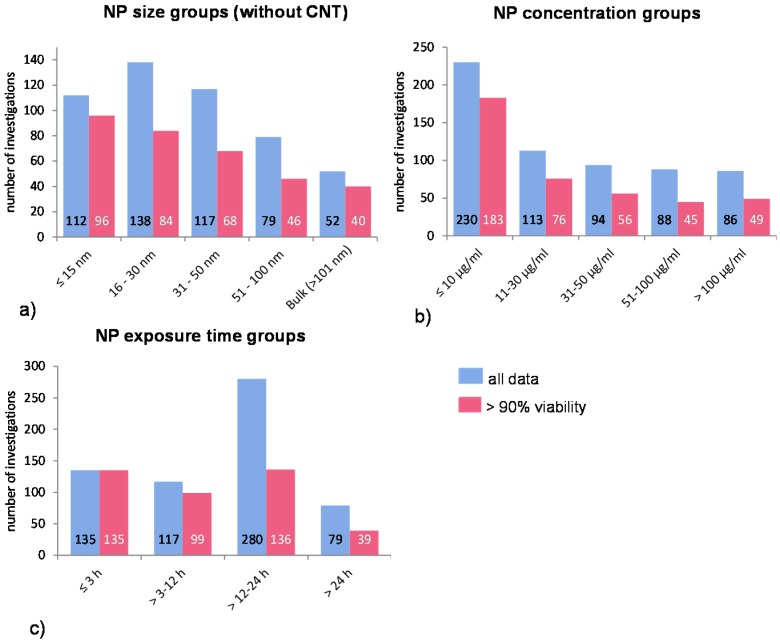
Distribution of all investigations according to NP (**a**) size- (without CNT data); (**b**) concentration-; and (**c**) exposure time groups. The blue bars represent the mean ROS ratios of all data, and the red bars the ROS ratios of cells with more than 90% viability. Data are illustrated as mean, and numbers inside the bars represent the number of investigations.

In particular, it has been shown that the NP uptake mechanisms seem to be NP size dependent [26]. Furthermore, there are different molecular mechanisms by which internalization takes place. The chosen size-groups consider these mechanisms in order to show possible mechanistic dependent size related NP uptake if there is any (Figure 1a).

The NP concentration groups are chosen according to very low, low, medium, high, and very high concentrations. Many investigations have used very high and, hence, not relevant concentrations of NP (above 50 μg/mL); thus, the cell survival rate was either not measured or it was below 90% (Figure 1b).

A very common exposure time for investigating phagocytic uptake is around 1 h. Accordingly, the first time period is up to 3 h, the next period is 3–12 h, and so on (Table 2). Extended exposure times can lead to intracellular accumulation of NP which in turn can influence the result by the induction of other intracellular processes (e.g., induction of apoptosis by NP overload leading to cell death and increased ROS release). However, since longer exposure times were employed in many studies, additional groups have been included to detect any possible influence of this experimental condition (Figure 1c).

In summary, ROS generation and cell viability were chosen as a group of biological endpoints. Two data sets were considered, *viz.* “all data” and “>90% viability” whereby 67% of “all data” showed >90% viability. We considered 66 publications and extracted 611 investigations, which were performed with 43 different cell types. The groupings of NP size, concentration and exposure time were chosen either according to cellular processes or arbitrarily.

### 2.4. Data Analysis of Selected NP

#### 2.4.1. Silicium Dioxide NP (SiO_2_-NP)

For the analysis of nano-silica, 16 publications were selected and 108 investigations (data points) were obtained (“all data”). Seventy-three percent of these belonged to the “>90% viability” group. Out of the 16 publications, only three reported the use of a positive control and four did not report the physicochemical properties of the used NP. The distinction between amorphous and crystalline nano-silica was made in six studies of which five employed amorphous silica in 27 investigations. This lack of information is noteworthy because it is reported that the crystalline form is more toxic while little is known about the amorphous form. However, Constantini *et al.* [27] compared the effects of both forms of nano-silica on various cell types and detected similar degrees of cytotoxicity, but also noted that macrophages reacted more strongly to nano-silica exposure than non-phagocytic cells. Professional phagocytic cells were used only in two publications (24 investigations) [8,28], and the remaining employed various human, murine or rat cell lines.

**Figure 2:**
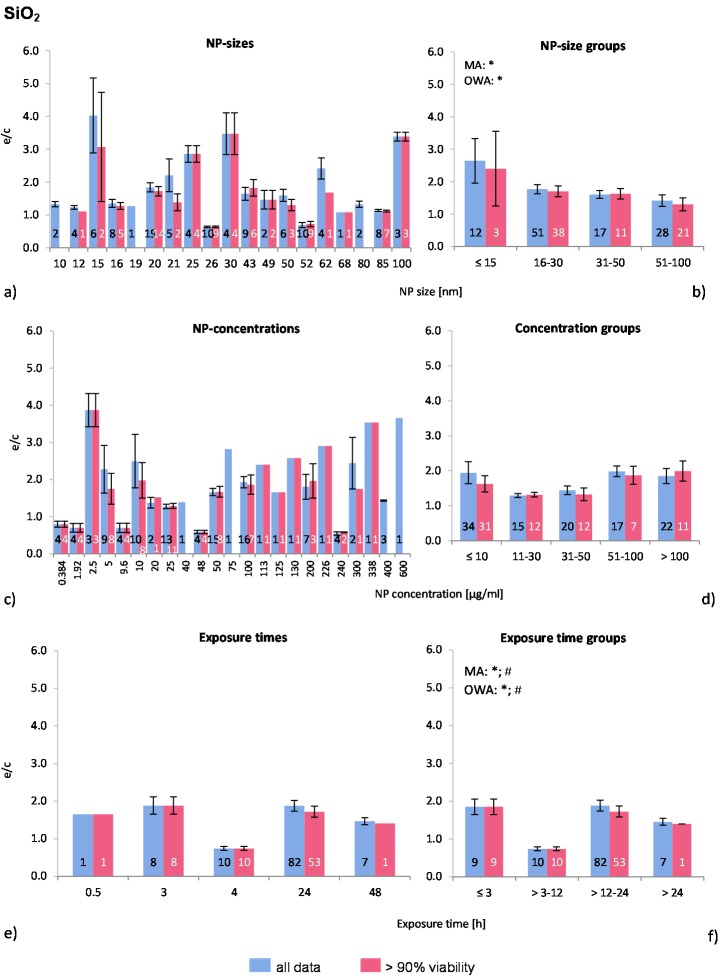
The effect of NP-size, concentration and exposure time on ROS ratios after exposure to SiO_2_-NP. The blue bars represent the mean ROS ratios of all data, and the red bars the ROS ratios of cells with more than 90% viability. Data are illustrated as mean ± SEM and numbers inside the bars represent the number of investigations. Mean ROS ratios as a function of (**a**) NP-size, (**c**) NP-concentration and (**e**) exposure times are shown. Multiple Regression Analysis (MRA) was used to identify correlations between the independent variables NP size, concentration or exposure time and the dependent variable ROS ratio; (**b**,**d**,**f**) show the respective groupings that were analyzed with multi-factor ANOVA (MA) for statistical significant inhomogeneity for three variables, one-way ANOVA (OWA) for one variable, and the modified post-hoc Tukey test for unequal n for group differences (*p* < 0.05 for * all data and # for >90% viability).

The distribution of the NP sizes was relatively even in the selected publications, as well as the applied NP concentrations, which, however, covered very large ranges. Almost 80% of all investigations were performed for 24 h (Figure 2a,c,e).

The Multiple Regression Analysis (MRA) statistical analysis test was performed for the whole data set considering the NP-size, concentration, and exposure time, and with the ROS ratio as the dependent variable. There was no correlation between any of these factors and ROS release.

Regarding the NP size groups (Figure 2b), the multi-factor ANOVA (MA) and one-way ANOVA (OWA) analyses detected significant inhomogeneity between the selected groups for “all data”. The modified Tukey test for unequal and did not support these findings. Figure 2b shows a slight decrease in ROS generation with increasing NP size, where the ≤15 nm group is significantly different from the other groups (ANOVA). Also, the percentage of investigations in the “>90% viability” data set is rising with larger NP size, indicating higher toxic effects of smaller NP. Out of the eight publications which tested more than one NP-size, six reported a size-dependent decrease of ROS release [9,10,29,30,31,32]. Our analysis supports these findings.

The data of the NP concentrations (Figure 2c) shows highly diverging mean values below NP concentrations of 10 μg/mL, with extremely high ROS ratios originating from one study by (Gong *et al.* [9]). In contrast, very low values were found in the study by Aranda *et al.* [8]. The number of investigations in the data set “>90% viability” is decreasing compared to “all data” with increasing NP concentrations (<10 μg/mL: 91%; 11–30 μg/mL: 80%; 31–50 μg/mL: 60%; 51–100 μg/mL: 41%; >100 μg/mL: 50%), suggesting a NP-concentration dependent cytotoxicity.

The NP concentration groups are statistically similar (Figure 2d). However, almost all publications reported a NP concentration-dependent generation of oxidative stress and reduction of cell viability [9,28,29,30,31,32,33,34,35,36,37]. Lactate dehydrogenase levels (LDH) were measured frequently also in combination with ROS whereby increasing LDH-levels were detected. LDH is an indicator for membrane damage and increased levels could indicate that the DCF probe could leak out and compromise the DCF-assay. In general, the NP concentrations employed are rather high considering the reported cytotoxicity of 10 μg/mL in HepG2 cells, using 15 nm NP and an EC_50_ of 50 μg/mL in A549 cells [38].

The exposure time groups (Figure 2f) resulted in statistically inhomogeneous sets for the >3–12 h by MA and OWA. Data were extracted from only one publication for this time point [8]. As most investigations were performed at 24 h exposure and only three studies [28,29,35] examined time dependent cell viability, the outcome of this comparative approach is not supported by the findings of individual publications. It seems likely that exposure time is not a determining factor for SiO_2_-NP induced toxicity, but without further investigations this outcome is not conclusive.

In summary, the presented comparative analysis for SiO_2_-NP reflects the findings of individual publications and a better insight is given by combining these. This can be observed for NP size groups, as the data analysis shows that the ROS release correlates with nano-SiO_2_ size. There is a statistically non-significant correlation between NP concentration and ROS release. Exposure time does not seem to influence the ROS generation rates.

#### 2.4.2. Titanium Dioxide NP (TiO_2_-NP)

Eleven publications fit the selection criteria and were analysed. The data compilation resulted in 75 investigations in total and in 49 data points for the “>90% viability”. The physicochemical characterization of the employed NP is rather scarce—only five studies stated whether the employed NP were of anatase or rutile form. Almost all experiments were performed with murine or human cells and, in two publications, rat cells were employed. Professional phagocytic cells were used in 49 investigations. Positive controls were used in three publications.

**Figure 3:**
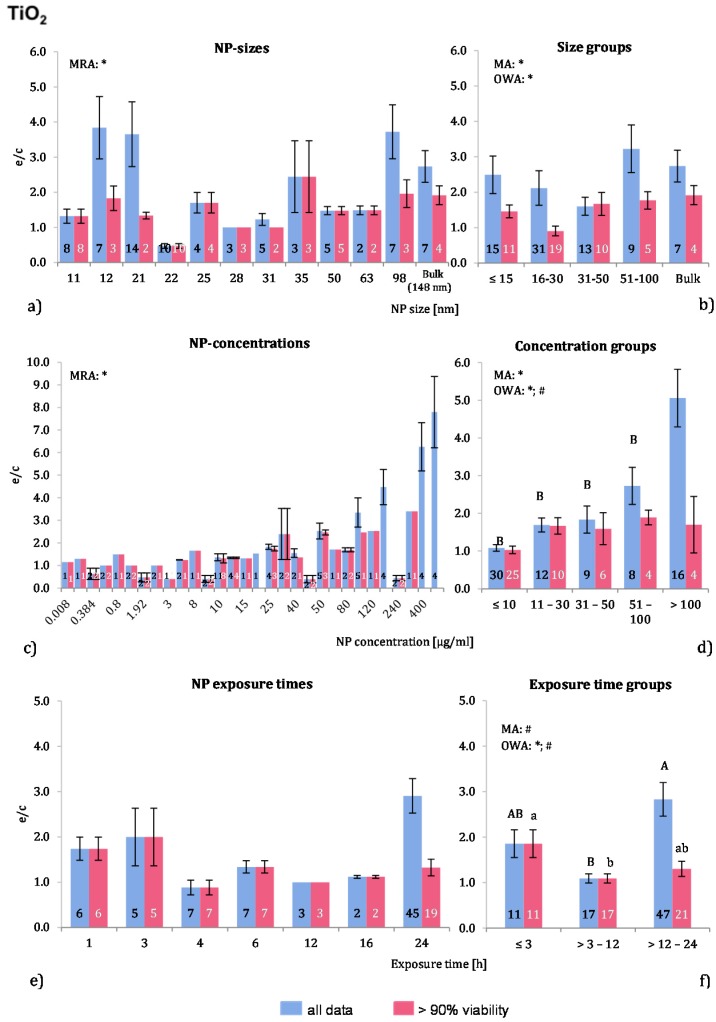
The effect of NP-size, concentration and exposure time on ROS ratios after exposure to TiO_2_-NP. Data are illustrated as mean ± SEM and numbers inside the bars represent the number of investigations. Mean ROS ratios as a function of (**a**) NP-size, (**c**) NP-concentration and (**e**) exposure times are shown. Multiple Regression Analysis (MRA) was used to identify correlations between the independent variables NP size, concentration or exposure time and the dependent variable ROS ratio; (**b**,**d**,**f**) show the respective groupings that were analyzed with multi-factor ANOVA (MA) for statistical significant inhomogeneity for three variables, one-way ANOVA (OWA) for one variable, and the modified post-hoc Tukey test for unequal n for group differences. The bars labelled “a” or “A” *etc.* are statistically different from “b” or “B” but not from “ab” or” AB”. *p* < 0.05 for * all data and # for >90% viability.

Almost 79% of the applied NP were below 50 nm in size. Sixteen investigations employed larger NP and in seven of these 148 nm NP were used. Although the ROS ratios of the raw data are quite fluctuating (Figure 3a), the MRA showed a significant correlation for the “>90% viability” data. Regarding the NP size groups, MA and OWA detected inhomogeneous groups among the “>90% viability”, but not for “all data”. The post hoc test did not support these findings. In the group with NP sizes of 16–30 nm, all 10 investigations with a NP size of 22 nm were derived from one publication [8], which apparently reduces the mean ROS ratio of the NP size group 16–30 nm to such an extent that it is statistically different from the others.

One publication tested three different TiO_2_-NP sizes on the same cell type [11] and observed a slightly higher ROS level with an NP size of 21 nm than with 12 nm or 98 nm. The same study also compared rutile and anatase forms and reported a less toxic response of the rutile TiO_2_-NP.

The ROS ratios were rising with increasing NP concentrations up to extreme values at very high concentrations (400 mg/mL) (Figure 3c). There was a correlation for “all data”, which was detected by MRA. In the grouping approach, MA and OWA analyses revealed a concentration dependent increase in ROS release (Figure 3d). The “all data” group with >100 µg/mL was significantly different from the other groups. There was a concentration dependent increase in ROS release in the two lowest concentration groups among the “>90% viability” groups, but concentrations above 30 μg/mL did not generate stronger responses. Most of the analysed publications reported a dose dependent increase in ROS levels [11,39,40,41,42,43,44,45], with two exceptions [8,46]. For the “all data” groups, the presented approach showed similar findings.

More than 60% of all investigations used 24 h exposure time, but 55% of these data points were obtained with cell viability below 90% (Figure 3e,f). Significant differences between the three time groups for both data sets were found using MA, OWA and the Tukey tests. After an increased ROS generation below 3 h, the ROS ratios are close to 1 during the 3–12 h exposure time span. Longer exposure times display similar results for the “>90% viability” but increased ratios for “all data”. In short, when viable cells are considered, ROS release seems to be decreasing with longer exposure periods.

**Figure 4:**
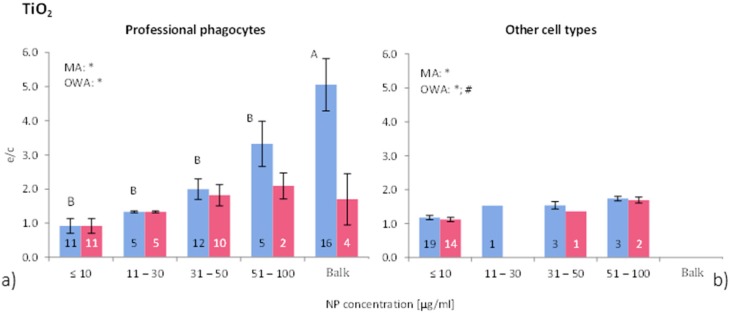
ROS ratios in (**a**) phagocytic cells, and (**b**) non-phagocytic cells after exposure to different concentrations of TiO_2_-NP. See Figure 2 for further details. The bars labelled “A” or “B” are statistically different. *p* < 0.05 for * all data and # for >90% viability.

This finding reflects the outcome of the investigations performed by Xia *et al.* [47] who observed, after an initial increase in ROS release, a drop over time in macrophages, but not in epithelial cells, which did not respond to NP exposure. Therefore, we performed a separate analysis in order to differentiate between professional phagocytes and non-phagocytic cells. Five studies employed professional phagocytes in their investigations of ROS release of TiO_2_-NP (49 investigations; 65% of all investigations) [8,11,43,44,47]. Statistically significant differences between the exposure time groups were detected by MA, OWA and the Tukey test. Figure 4 shows the differences in ROS release due to NP concentration for phagocytic and all other cells. Overall, the ROS ratios of phagocytic cells without consideration of group classification, is 2.2 times higher for “all data” and 1.2 times for “>90% viability”. Non-phagocytic cells show weak to no response to TiO_2_-NP exposure. This result suggests that TiO_2_-NP are not as toxic to non-phagocytic cells compared to other metal oxide NP, which also was reported by e.g., [45,46]. On the other hand, professional phagocytes react more strongly to NP exposure by inducing an oxidative stress during phagocytosis, which is a physiological response of phagocytes [48,49].

According to the analysed data, the significant effect of TiO_2_-NP exposure on ROS release is correlated to exposure time and NP concentration but not to NP size. The increase in ROS release is attributable to the use of phagocytic cells, since a refined data analysis revealed that professional phagocytic cells generate higher ROS ratios than other cell types. The crystalline form of TiO_2_-NP might be crucial for ROS generation as well, but due to the lack of the physicochemical descriptions of the employed NP a distinction between the different forms was not possible to perform.

#### 2.4.3. Zinc Oxide NP (ZnO-NP)

ZnO-NP was selected to be analysed here because of its high toxicity and solubility. Seventeen publications were selected for the present analysis. In total, 166 investigations were extracted from the publications but only 82 investigations remained in the “>90% viability” data set. Most experiments were executed on a variety of human cells and to a lesser degree on murine cells. One publication employed rat cells and, in one large study, fish cells were treated with ZnO-NP. Regarding the quality of the studies, only three articles presented results of positive controls (18% of the selected publications). The shapes of the ZnO-NP are diverse and were described as rods, spheres, elongated, polygonal sheet shape, and wurzite crystalline structure. For comparison purposes, the largest dimension was taken as the NP size (e.g., for rods described with thickness and length, the length was considered as the NP size). Although Gratton *et al.* [19] showed a preferred uptake of rod-like shapes over spherical ZnO-NP, the shape is often not mentioned when the toxicity of ZnO-NP is examined.

**Figure 5:**
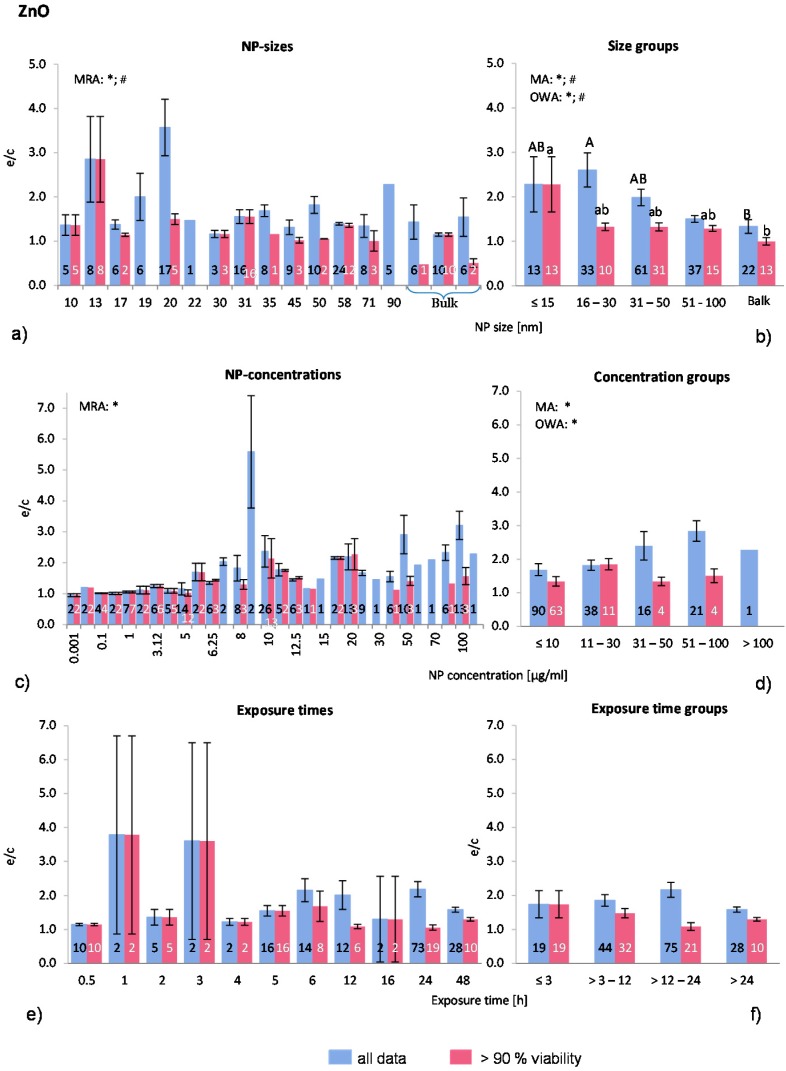
The effect of NP-size, concentration and exposure time on ROS ratios after exposure to ZnO-NP. Data are illustrated as mean ± SEM and numbers inside the bars represent the number of investigations. Mean ROS ratios as a function of (**a**) NP-size, (**c**) NP-concentration and (**e**) exposure times are shown. Multiple Regression Analysis (MRA) was used to identify correlations between the independent variables NP size, concentration or exposure time and the dependent variable ROS ratio; (**b**,**d**,**f**) show the respective groupings that were analyzed with multi-factor ANOVA (MA) for statistical significant inhomogeneity for three variables, one-way ANOVA (OWA) for one variable, and the modified post-hoc Tukey test for unequal n for group differences. The bars labelled “a” or “A” *etc.* are statistically different from “b” or “B” but not from “ab” or” AB”. *p* < 0.05 for * all data and # for >90% viability.

The distribution of the employed NP-sizes is relatively balanced, as Figure 5a shows. There are 22 investigations with NP over 100 nm. MRA analysis revealed statistically significant correlations between both data sets. Statistically significant differences between the groups were detected when applying the MA, OWA and the Tukey tests. The largest particles (>100 nm) are different from the smallest NP with a diameter of ≤15 nm in the “>90% viability” data set (Figure 5b). In “all data”, the group with >16–30 nm has a higher ROS ratio, however, otherwise the ROS release decreases with decreasing NP size. Only one study [50] tested different NP sizes (19 nm; 71 nm; 108 nm and 342 nm) and found the highest ROS release at the smallest NP size exposure, which is consistent with the outcome of the present study.

About three quarters (77%) of all investigations were performed with NP concentrations below 30 μg/mL (Figure 5c,d). MRA analysis shows a dose dependent increase of ROS generation for the “all data” category. The NP concentration groups of “all data” are also significantly different according to MA and OWA in a concentration dependent manner. As for the “>90% viability”, the ROS releases increase slightly until the concentration of 30 μg/mL is reached, but drops afterwards.

The expanding relative discrepancy between the two data sets is noteworthy. In the “>90% viability” data set, the percentage of investigations is declining rapidly with increasing NP concentration (≤10 μg/mL: 70%; 11–30 μg/mL: 29%; 31–50 μg/mL: 25%; 51–100 μg/mL: 19%; >100 μg/mL: 0%), indicating a high cytotoxicity at high NP concentrations. The publications that tested different NP concentrations all reported a dose-dependent reduction of cell viability with the concomitant rise in ROS generation. The data suggest a dose dependency which is restricted to up to 30 μg/mL NP concentrations.

Due to the large amount of investigations at low ZnO-NP concentrations, the group ≤10 μg/mL was divided into three subgroups for a refined analysis. The results were not statistically different from the presented group classification (data not shown).

Exposure time does not influence the ROS release although there is a trend towards that mean group values of “>90% viability” indicate a small drop after 3–12 h exposure (Figure 5f), suggesting a decrease in ROS release with longer exposure times. This is in contrary to the “all data” groups, where no such effect is seen. Similar to the NP concentrations, the percentage of investigations in the “>90% viability” data set is declining when compared to the “all data” set. This could be the reason for the reported time dependency by some authors [51,52,53,54] detecting rising ROS ratios with longer exposure time.

Taken together, the ROS release after exposure to ZnO-NP is NP size and concentration dependent, but most likely not related to exposure time. However, our approach reflects the outcome of reported behaviours of cells for “all data”.

#### 2.4.4. Copper Oxide NP (CuO-NP)

Despite of the known toxicity of CuO, there are surprisingly few publications available regarding the effects of this material in nano form on ROS release *in vitro*. In this study, eight publications were detected and analysed leading to a data assembly of 40 investigations of which 28 remained in the “>90% viability” data set. One study [55] did not mention the physicochemical properties of the applied NP, but all others gave at least some information. Positive controls were lacking in all studies. Human and murine cell lines were used in all studies with exception of one case [56], where primary murine phagocytes were employed.

**Figure 6:**
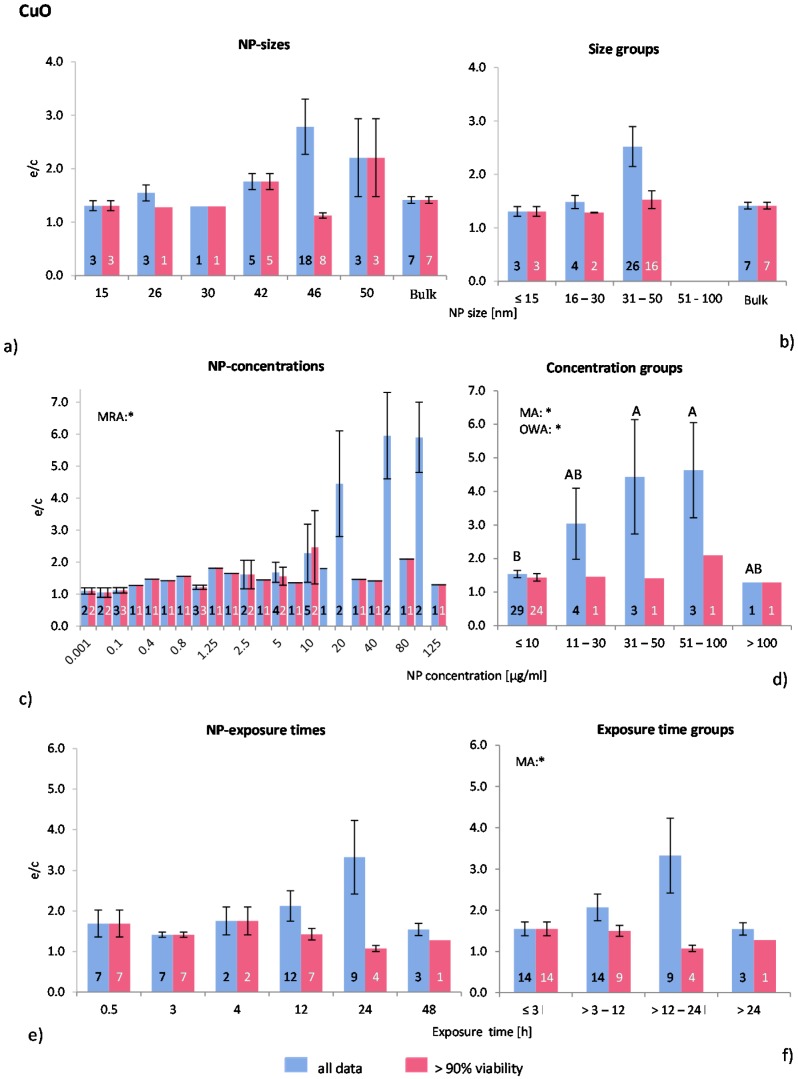
The effect of NP-size, concentration and exposure time on ROS ratios after exposure to CuO-NP. Data are illustrated as mean ± SEM and numbers inside the bars represent the number of investigations. Mean ROS ratios as a function of (**a**) NP-size, (**c**) NP-concentration and (**e**) exposure times are shown. Multiple Regression Analysis (MRA) was used to identify correlations between the independent variables NP size, concentration or exposure time and the dependent variable ROS ratio; (**b**,**d**,**f**) show the respective groupings that were analyzed with multi-factor ANOVA (MA) for statistical significant inhomogeneity for three variables, one-way ANOVA (OWA) for one variable, and the modified post-hoc Tukey test for unequal n for group differences. The bars labelled “a” or “A” *etc.* are statistically different from “b” or “B” but not from “ab” or” AB”. *p* < 0.05 for * all data and # for >90% viability.

The statistical analysis for NP size and size groups gave no significant results (Figure 6a,b). Most experiments were performed within the NP size group 31–50 nm where the highest effects were shown for “all data” only. The statistical analyses, however, showed that NP size does not have any influence on ROS release.

Almost three quarters of all investigations were performed with NP concentrations below 10 μg/mL, however, huge ROS ratios were detected in “all data” at high NP concentrations (Figure 6c). MRA, MA and OWA detected significant correlations between ROS ratios and NP concentrations in “all data”. The Tukey test showed that the NP concentration group of >10 μg/mL is statistically different from the groups 31–50 μg/mL and 51–100 μg/mL (Figure 6d). ROS ratios rise with increasing NP concentrations, but both the number of investigations and the cell viability values are low in these groups.

Since the majority of the investigations were performed at NP concentrations ≤10 μg/mL, this group was subdivided into three subgroups (≤1, 2–5 and 6–10 μg/mL) for a refined analysis. A slight increase in ROS release for “>90% viability” up to 10 μg/mL was identified, but this was statistically insignificant. Similar effects were detected for “all data” (data not shown). Interestingly, all publications examined here reported dose dependent cytotoxicity and ROS generation.

The original data for exposure time show no statistical differences according to MRA (Figure 6e). However, significant inhomogeneity between the exposure time groups for “all data” was detected by using MA, but not by OWA. Figure 6f shows a rise in ROS ratios up to an exposure time of 24 h followed by a drop afterwards. Regarding the “>90% viability” data, no statistically significant difference was detected.

In summary, the size and exposure time of CuO-NP are not seen to influence the *in vitro* ROS generation. However, ROS generation seems to be NP concentration dependent at very low concentrations (>10 μg/mL). In spite of this, since available data are limited, this analysis is not delivering a clear result.

#### 2.4.5. Nanoceria (Ce_2_O_3_/CeO_2_-NP)

Only eight publications fulfilled the selection criteria for the present study, providing 109 data points (102 for “>90% viability”). Seven different cell types were used, of which two were professional phagocytes. In total, 47 investigations were performed with such cells. More than 99% of all investigations used human cells complemented by murine cells. Three publications included positive controls.

**Figure 7:**
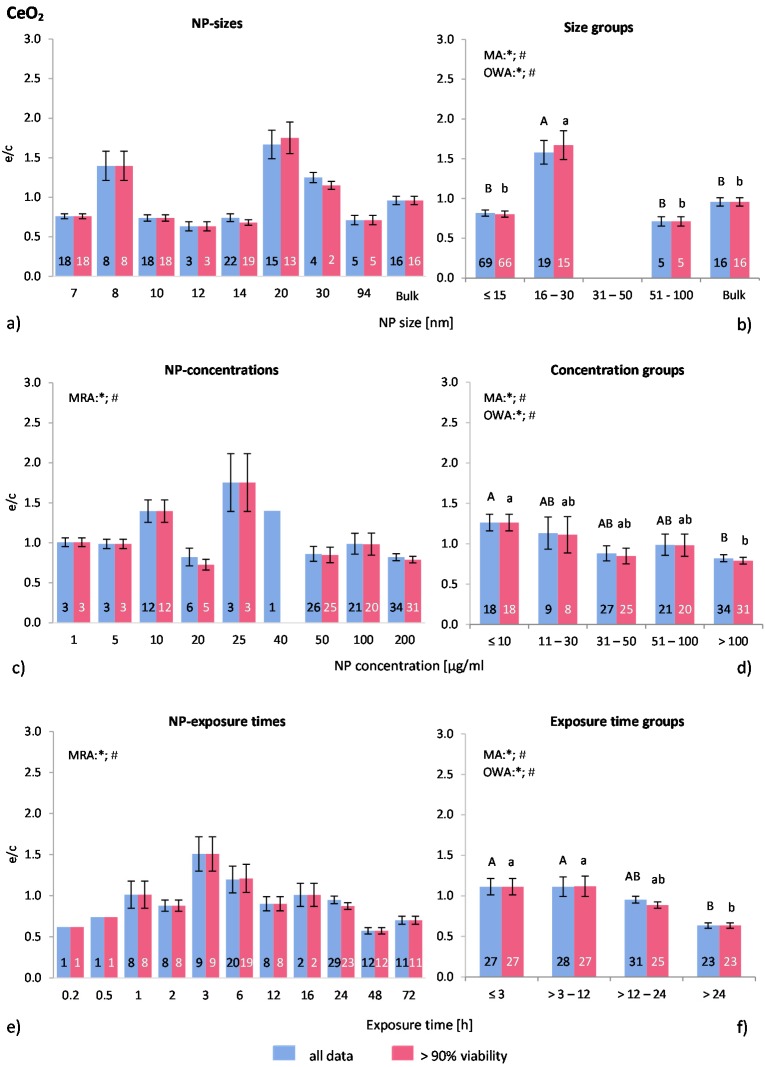
The effect of NP-size, concentration and exposure time on ROS ratios after exposure to CeO_2_-NP. Data are illustrated as mean ± SEM and numbers inside the bars represent the number of investigations. Mean ROS ratios as a function of (**a**) NP-size, (**c**) NP-concentration and (**e**) exposure times are shown. Multiple Regression Analysis (MRA) was used to identify correlations between the independent variables NP size, concentration or exposure time and the dependent variable ROS ratio; (**b**,**d**,**f**) show the respective groupings that were analyzed with multi-factor ANOVA (MA) for statistical significant inhomogeneity for three variables, one-way ANOVA (OWA) for one variable, and the modified post-hoc Tukey test for unequal n for group differences. The bars labelled “a” or “A” etc. are statistically different from “b” or “B” but not from “ab” or” AB”. *p* < 0.05 for * all data and # for >90% viability.

The distribution of the NP size of the applied nanoceria in the respective publications is unbalanced—64% of the investigations employed NP sizes below 15 nm, followed by NP sizes between 16 and 30 nm (Figure 7a). By using MRA, no correlation was found between NP size and ROS release. The highest ROS ratios were reported by Mittal *et al.* [57] using 20 nm NP. This study showed elevated ROS levels in A549 human adenocarcinoma lung epithelial cells. Horie *et al.* [58] investigated the ROS levels in epidermal keratinocytes with similar results. The second highest ROS ratios were generated by 8 nm NP in a study on mouse macrophages and human epithelial cells where only the macrophages responded with an increased ROS release [47]. The other investigations using phagocytic cells showed similar antioxidative effects (at the same magnitude) as with the other cell types.

Statistically significant differences are shown in the NP size groups according to MA, OWA and Tukey tests (Figure 7b). NP with a size of 16–30 nm generated statistically significant higher ROS ratios than all other groups.

The applied ranges of NP concentrations are between 1 and 200 μg/mL (Figure 7c). According to the MRA test both data sets significantly correlated with this variable. The groups illustrate a NP concentration dependent effect even more obviously as shown in Figure 4d. Below 10 μg/mL ROS ratios are around 1.3, followed by a steady drop to 0.8 at higher concentrations. All three statistical tests (MRA, MA, OWA) as well at the Tukey test confirmed these findings for both data sets. A refined group classification did not reveal statistically different results (NP concentration group ≤10 μg/mL was split into ≤5 μg/mL and 6–10 μg/mL; data not shown).

Among the investigated studies, only Park *et al.* [59] and Mittal *et al.* [57] detected a dose dependent increase in ROS release, whereas Ting *et al.* [60] described a dose dependent decrease. Based on the available material, our approach showed a statistically significant inverse relationship between NP concentration and ROS release.

Within the raw data of exposure times for both data sets, significant correlations between the variables were detected using MRA (Figure 7e). The exposure time frame among the investigations was evenly distributed between 3 min and 72 h. The exposure time groups show that short exposure times (≤3 h) generated almost no ROS release, similar to the 3–12 h group (Figure 7f). These two groups are significantly different from the >24 h group, which has a ROS ratio of 0.6. MA, OWA and the post-hoc test indicated significant differences among the groups. Because the raw data in Figure 7e indicate an increase in ROS generation until 3 h exposure time (from 0.6 to 1.5) this group was analysed in more detail by splitting it into two subgroups. However, the ROS ratios generated below 1 h of exposure were not statistically different from the mean ratios between 1 and 3 h (data not shown). Similar to the NP concentration groups, the statistical analysis show also a time dependent antioxidative effect of nanoceria.

Two major findings are notable from this analysis: First, nanoceria has an antioxidative effect, as it was reported by Karakoti *et al.* [61] and many others, which is caused by the two oxidation states of Cerium (Ce^3+^/Ce^4+^). With higher NP concentrations and longer exposure time more radicals are scavenged and thus the ROS level is reduced. Secondly, the variation between distinct investigations is relatively low.

The cell viability in the selected publications was generally very high or not measured at all. Only Mittal *et al.* [57] and Park *et al.* [59] reported reduced cell viability in a time, concentration and size dependent manner. Lord *et al.* [7] detected an increased cell viability compared to control. All other examined publications found no changes regarding cell viability.

In summary, nanoceria act as an antioxidant in a concentration and time dependent manner, which was verified by several statistical tests. Significant differences for NP size groups were identified as well, but other parameters than the ones considered here seem to influence the results more than the NP size. Furthermore, exposure to nanoceria had no adverse effect on cell viability. In general, the presented analysis strengthens the information regarding nanoceria toxicity and supports the findings of individual publications.

#### 2.4.6. Carbon Nano Tubes (CNT)

Seventeen studies were selected that provided 113 data points, of which 75 were categorized as “>90% viability”. No distinction was made between single wall CNT (SWCNT) and multi wall (MWCNT), which were employed 14 times and eight times, respectively. In all publications, the used CNT were purchased, and most of the authors stated the companies’ name. Information about physicochemical properties were limited to TEM or SEM analysis. The CNT surface area and the zeta potential were stated twice, and information about the shape was given in four publications. Seven publications used positive controls. Regarding viability tests, one author was referring to a previous study where no cytotoxicity was detected [62], others presented contradicting results of various tests (e.g., [63,64]). Although possible interactions between tetrazolium salts and CNT are known [25], the MTT assay was still routinely employed as a viability test for CNT exposed cells.

Altogether 12 cell types of mostly human origin were employed, of which, two were professional phagocytes (macrophages) derived from mouse and rat, respectively. These cell types were used in two publications [63,65] containing 22 investigations. Chen *et al.* [65] compared human alveolar epithelial cells (A549 cells) with murine macrophages (RAW264.7 cells) and identified similar responses in ROS release and cytotoxicity. In contrast, Murphy *et al.* [21] examined inflammatory responses in human mesothelial cells and human monocytes and observed higher inflammation potential in monocytes/macrophages, attributed to frustrated phagocytosis.

The pooling and choice of groups for CNT size is more difficult than for spherical NP, because CNT-sizes are described by two parameters (length and thickness) instead of one. In general, here CNT were first classified according to length (≤10 μm and >10 μm) and then according to thickness into six groups (Table 2). The 10 μm measure is generally considered as the length of a particle which can be internalized by a cell.

**Figure 8:**
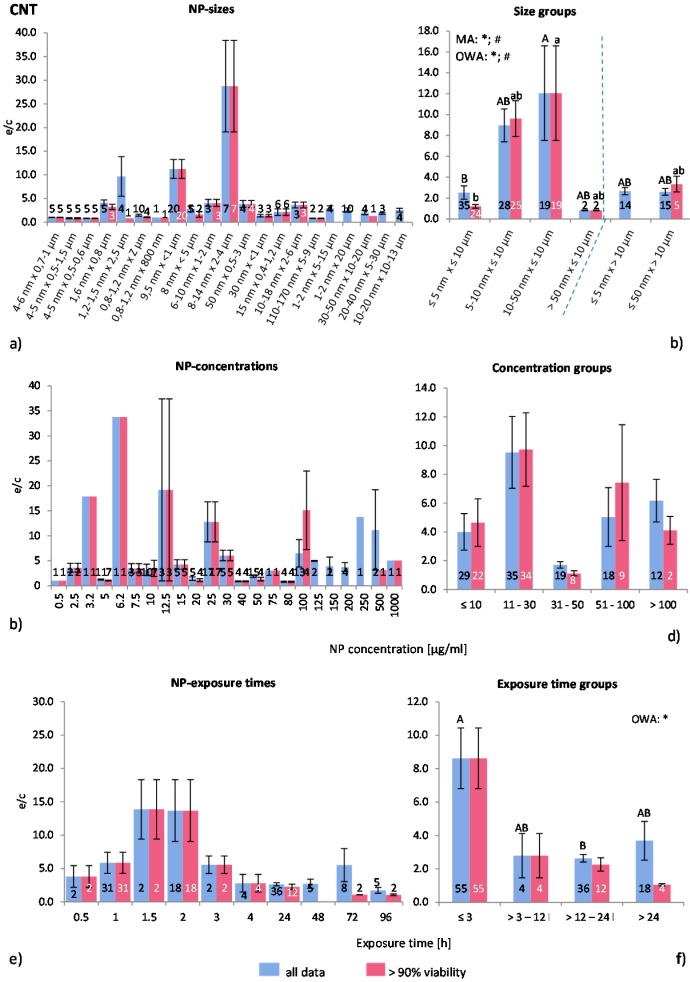
The effect of NP-size, concentration and exposure time on ROS ratios after exposure to CNT-NP. Data are illustrated as mean ± SEM and numbers inside the bars represent the number of investigations. Mean ROS ratios as a function of (**a**) NP-size, (**c**) NP-concentration and (**e**) exposure times are shown. Multiple Regression Analysis (MRA) was used to identify correlations between the independent variables NP size, concentration or exposure time and the dependent variable ROS ratio; (**b**,**d**,**f**) show the respective groupings that were analyzed with multi-factor ANOVA (MA) for statistical significant inhomogeneity for three variables, one-way ANOVA (OWA) for one variable, and the modified post-hoc Tukey test for unequal n for group differences. The bars labelled “a” or “A” *etc.* are statistically different from “b” or “B” but not from “ab” or” AB”. *p* < 0.05 for * all data and # for >90% viability.

Figure 8a demonstrates the ROS ratios for the collected raw data as dependent on size. The ROS ratios fluctuate considerably with large SEM. The MRA test showed no significant correlations between the variables. The groups show, however, by using MA, OWA and the Tukey test, that there are statistically significant differences between the NP size groups (Figure 8b). Exposure to CNT longer than 10 μm leads to a lower average ROS release than shorter CNT, possibly because of the ongoing internalization mechanism. However, CNT with >50 nm thickness and <10 μm length show much lower ROS generation than the slightly thinner CNT. The latter result was extracted from two investigations only, and, therefore, the relevance of this outcome has to be tested using more data.

The data and the groups related to CNT concentration show extremely variable ROS ratios (Figure 8c,d). The statistical tests did not identify any significant differences, although the majority (14 out of 17) of the examined publications reported a dose dependent increase in ROS release. This indicates that other parameters, such as surface modifications [66], affect this grouping classification, since reactivity and toxicity of individual types of CNT are differing largely.

Regarding exposure time (Figure 8e), ROS ratios were increased up to an exposure time of 1.5 h followed by a drop after 2 h. There was no correlation between any of the variables detected with the MRA although the exposure time groups (Figure 8f) show a time dependent decrease in ROS ratios, according to the OWA and Tukey test for “all data”. At exposure times below 3 h, the ROS ratios are relatively high, followed by declining values at longer times. This suggests that there is an inverse correlation between ROS release and CNT exposure time. However, several studies performing time-dependent tests reported that ROS generation increases with elongated exposure time [65,67,68].

Altogether, ROS ratios generated by CNT are very variable. Statistically significant correlations were identified for NP size and exposure time. The CNT pooling gave inconclusive results, suggesting that other factors than the ones analysed here are important. The analysed amount of data seems to be too small for the analysis, thus no conclusions could be drawn. Further analysis with more data could lead to a better understanding of CNT toxicity, considering that CNT is possibly a group of NP by itself.

## 3. Experimental Section

### 3.1. Selection of NP

We selected six different NP for the analysis. These were SiO_2_, TiO_2_, ZnO, CuO, CeO_2_/Ce_2_O_3_ NPs, and carbon nanotubes (CNT) for the following reasons:

TiO_2_ and SiO_2_ NPs are well investigated materials in both nano- and in bulk form; despite their reactive surface, TiO_2_ NP are considered to be non-toxic; both NP have high production volumes worldwide [69,70] and are present in a wide range of consumer products. These NP might reflect the suitability of the pooling approach.

ZnO- and CuO-NP are toxic, reactive and soluble materials; cytotoxicity has been detected at already very low doses (e.g., [45,71]). Here, these EMNs aim to represent soluble metal oxide NP.

CeO_2_ acts presumably as ROS scavenger and is considered as exhibiting low toxicity *in vitro* (e.g., [59]). Since the antioxidant effect of this NP is controversial, our approach is expected to elucidate this matter.

CNT exists in many different forms, with a variety of surface modifications and different size parameters (e.g., [72]); if CNT expresses asbestos-like features they behave like asbestos and are toxic; other forms are considered as non-toxic. The CNT data were tested here to clarify the limits of the present approach.

### 3.2. Selection Criteria for Publications

In the present study, we used peer reviewed publications collected from PubMed [73] and Sciencedirect [74] and filtered according to the following selection criteria:

*In vitro* experiments analysing the selected NP uptake via ROS production. ROS release is a frequently used measure for NP testing; therefore, a wide range of publications is available. It reflects the amount of oxidative stress that is generated by the NP uptake and, consequently, it is assumed that it indicates the degree of NP hazard.

To enable a reasonable comparison of the studies, only those publications were considered in which the tested NP concentration was stated as mass units per volume.

For the comparative analysis, only publications were considered that used the 2′,7′-dichlorofluorescin diacetate (DCF)-assay for ROS measurement.

Experiments should be performed in at least three independent experiments and at the probability value of *p* < 0.05 at minimum.

To ensure the actuality of the data, publications after 2008 were considered.

### 3.3. Software-Assisted Data Acquisition and Compilation

The majority of the published data were presented in graphs without providing the values of single measured data points. For a possible and comparable data analysis, all data points were converted into a common measure by using the graph-digitizing software GetData Graph Digitizer version 2.26 (www.getdata-graph-digitizer.com). To access the published data, the graphs of interest were copied, transformed into a jpeg-format, and processed with the GetData software. By this procedure, ratios of intracellular ROS release were generated for all data points. The GetData Graph Digitizer was not providing any information about accuracy.

The information extracted from the selected studies is available in the Supplement (see Table S1) containing the categories “NP characterization”, “quality of study” and “experimental parameters”. The NP characterization included the information given by the authors about NP suppliers, manufacturing procedure, and physicochemical parameters and characterization methods. For the category “quality of study”, information regarding the use of positive controls was collected and the realisation of the statistical selection criteria (experiments should be performed in three independent trials and at the probability value of *p* < 0.05 at minimum) was ensured. As relevant data for “experimental parameters”, cell type, origin species, NP concentration, exposure time, and the ROS detection method were collected. Furthermore, information about the “cell viability” was collected if given by the authors. As a minimum threshold of cell viability, 90% was arbitrarily chosen. ROS ratios for cell viabilities of 90% or above were assigned to “>90% viability”, all other data points were assigned to “all data”.

### 3.4. Data Processing and Classification into Groups

All data points with similar NP size, concentrations or exposure times were compiled to mean ROS ratios (experiment over control values: e/c). The numbers of investigations, which are different from the numbers of publications, are stated in the corresponding graphs. The specific group-ranges were chosen according to known toxicological and cell physiologic processes if possible, as described in the Introduction, otherwise arbitrarily (see Table 1).

### 3.5. Statistical Data Analysis

Since all obtained data are expressed as ROS ratios without a “control” value, different statistical methods were applied for data analysis using the software Statistika version 7.1 by statsoft (www.statsoft.de).

The Multiple Regression Analysis (MRA) is a correlation test that was used for each NP to obtain information about the relationship between the independent variables NP size, concentration and exposure time and the dependent variable ROS ratio.

The multi-factor ANOVA (MA) was used to test the statistically significant inhomogeneity between the groups considering all three influencing variables.

The one-way ANOVA (OWA) shows statistically significant inhomogeneity between the groups considering only one influencing variable.

The modified Tukey post-hoc test for unequal n was used to test statistically significant differences of the OWA, to identify the group differences.

## 4. Conclusions

The aim of this study was to test the applicability of a pooling approach for selected nanomaterials-induced published *in vitro* data in order to identify the toxic capacity of specific NP. Six different NPs were selected for the analysis. Two common biological endpoints (intracellular ROS and ROS production in cells with viability above 90%) were identified for a comparative approach. The correlations of the ROS ratios between exposed and control cells with different experimental parameters such as NP size, concentration, and exposure time were analysed.

Regarding NP-size, we showed that SiO_2_- and ZnO-NP induce decreasing ROS ratios with increasing NP-size, whereas the other investigated NP indicate inhomogeneous groups but with no clear correlation. The analysed NP-concentration groups (excluding CNT) point to increasing ROS ratios with increasing NP-concentrations, and only CeO_2_-NP is causing a decreased ROS level because of its scavenging effect. Interestingly, our data compilation shows that ROS ratios are increasing with the exposure time for “all data” but decreasing for “>90% viability”. Only CeO_2_-NP and CNT lead to decreasing ROS ratios over time.

In general, the applied method allows comparing information received from particular studies since:

- investigations were compared qualitatively;
- investigations were compared quantitatively enabling the application of statistical methods;
- technical and experimental inconsistencies that occur in individual studies are evened out;
- group-ranges regarding NP size and concentration are more representative than single unit values (e.g., 16–30 nm instead of 21 nm) for what the cells are exposed to (“seeing”).

The outcome of the pooling of the selected NP shows that this approach is applicable for the selected metal oxide NP, but might need reconsideration and a larger data set for e.g., CNT. For several analyses, statistically significant results were obtained, confirming the outcome of the majority of individual studies and the already known specific toxicities. In cases where the analysis did not show any expected correlation between a variable and the corresponding ROS ratios (e.g., size grouping for TiO_2_-NP and CuO-NP or time grouping for SiO_2_-NP), either the data amount was insufficient or other factors than the ones investigated in this study influence the ROS release. Such factors could be e.g., agglomeration/aggregation of the NP, or different reactivity of various crystalline forms, *etc.*

For a proper comparative analysis, a comprehensive description of the physicochemical parameters is needed as well as a large number of data. In the present study, data are limited for CuO-NP and CNT, and, thus, the power of the statistical analysis is weak. Additionally, CNT might already be a “group of NP” by itself since it is described at least by two parameters (length and thickness), and CNT is existing in many different variants. This needs further investigation.

The choice of intracellular ROS generation for the testing of NP-induced cell responses as an endpoint was appropriate. Therefore, this endpoint is valuable for toxicological investigations. However, this implies the assumption that the tested cells are viable, which was frequently not the case in the present selected data. Thus, it must be emphasized that ROS release and cell viability should always be tested in combination.

Significant differences between professional phagocytes and non-phagocytic cells were detected. Considering ROS production as the biological endpoint, it might be relevant to investigate both cell types in parallel. Further analyses of available data could probably provide answers to the cell type specificity of response.

Although the employed *in vitro* methods are commonly applied, many protocols are not standardized. Interestingly, it seems that the applied approach is balancing out the lack of standardization. This has, however, to be analysed in more detail before any firm conclusion can be drawn.

An extension of the present analysis by pooling more data, selecting more variables and different endpoint(s), using a more sophisticated computer based method, *etc.* would allow using this approach as a tool for comparative toxicity data evaluation. Furthermore, the present study shows that the use of NP size, concentration and exposure time alone are not always allowing clear statements regarding toxicity of a particular NP.

In the dose-effect paradigm, the mass is used as a dose metric, and, in the majority of all studies of NP toxicity, this parameter is applied. It is quite evident that this single dose parameter is not sufficient to describe the toxic effects of nanoparticles. Nevertheless, the majority of the publications state the applied concentration and exposure time. More useful specifications could be applied such as the surface area/unit, which would consider agglomeration [75], or the internalized mass [76], although the latter do not consider all relevant particle characteristics. Simkó *et al.* [75] proposed a dose metric model where the surface area is the main metric and the physicochemical properties are given different importance by weighting factors. The use of the NP surface area (size + concentration) as a function of exposure time could constitute more appropriate dose-response metrics.

In conclusion, the presented pooling and analysis of available data is a promising method for data evaluation and providing insight about NP induced toxicity *in vitro*. If more data are included, the power of the statistical analysis becomes stronger. Its potential is not limited to ROS release, and, thus, any other biological endpoint can be applied.

## Supplementary Materials

Supplementary materials can be found at http://www.mdpi.com/1422-0067/16/11/25954/s1.

## References

[1] Krug H.F. (2014). Nanosafety research—Are we on the right track?. Angew. Chem..

[2] Organisation for Economic Co-operation and Development (OECD) (2014). Guidance on grouping of chemicals. Series on testing and assessment. ENV/JM/MONO(2014).

[3] Arts J.H., Hadi M., Keene A.M., Kreiling R., Lyon D., Maier M., Michel K., Petry T., Sauer U.G., Warheit D. (2014). A critical appraisal of existing concepts for the grouping of nanomaterials. Regul. Toxicol. Pharmacol..

[4] Walser T., Studer C. (2015). Sameness: The regulatory crux with nanomaterial identity and grouping schemes for hazard assessment. Regul. Toxicol. Pharmacol..

[5] Oomen A.G., Bos P.M., Fernandes T.F., Hund-Rinke K., Boraschi D., Byrne H.J., Aschberger K., Gottardo S., von der Kammer F., Kuhnel D. (2014). Concern-driven integrated approaches to nanomaterial testing and assessment—Report of the nanosafety cluster working group 10. Nanotoxicology.

[6] Stone V., Pozzi-Mucelli S., Tran L., Aschberger K., Sabella S., Vogel U., Poland C., Balharry D., Fernandes T., Gottardo S. (2014). ITS-NANO—Prioritising nanosafety research to develop a stakeholder driven intelligent testing strategy. Part. Fibre Toxicol..

[7] Lord M.S., Jung M., Teoh W.Y., Gunawan C., Vassie J.A., Amal R., Whitelock J.M. (2012). Cellular uptake and reactive oxygen species modulation of cerium oxide nanoparticles in human monocyte cell line U937. Biomaterials.

[8] Aranda A., Sequedo L., Tolosa L., Quintas G., Burello E., Castell J.V., Gombau L. (2013). Dichloro-dihydro-fluorescein diacetate (DCFH-DA) assay: A quantitative method for oxidative stress assessment of nanoparticle-treated cells. Toxicol. In Vitro.

[9] Gong C., Tao G., Yang L., Liu J., He H., Zhuang Z. (2012). The role of reactive oxygen species in silicon dioxide nanoparticle-induced cytotoxicity and DNA damage in hacat cells. Mol. Biol. Rep..

[10] Li Y., Sun L., Jin M., Du Z., Liu X., Guo C., Li Y., Huang P., Sun Z. (2011). Size-dependent cytotoxicity of amorphous silica nanoparticles in human hepatoma hepg2 cells. Toxicol. Vitr..

[11] Zhang J., Song W., Guo J., Zhang J., Sun Z., Li L., Ding F., Gao M. (2013). Cytotoxicity of different sized TiO_2_ nanoparticles in mouse macrophages. Toxicol. Ind. Health.

[12] Jana N.R. (2011). Design and development of quantum dots and other nanoparticles based cellular imaging probe. Phys. Chem. Chem. Phys..

[13] Rejman J., Oberle V., Zuhorn I., Hoekstra D. (2004). Size-dependent internalization of particles via the pathways of clathrin- and caveolae-mediated endocytosis. Biochem. J..

[14] Sahay G., Alakhova D.Y., Kabanov A.V. (2010). Endocytosis of nanomedicines. J. Control. Release.

[15] Iversen T.-G., Skotland T., Sandvig K. (2011). Endocytosis and intracellular transport of nanoparticles: Present knowledge and need for future studies. Nano Today.

[16] Mao Z., Zhou X., Gao C. (2013). Influence of structure and properties of colloidal biomaterials on cellular uptake and cell functions. Biomater. Sci..

[17] Shang L., Nienhaus K., Nienhaus G.U. (2014). Engineered nanoparticles interacting with cells: Size matters. J. Nanobiotechnol..

[18] Kato H., Nakamura A., Takahashi K., Kinugasa S. (2012). Accurate size and size-distribution determination of polystyrene latex nanoparticles in aqueous medium using dynamic light scattering and asymmetrical flow field flow fractionation with multi-angle light scattering. Nanomaterials.

[19] Gratton S.E., Ropp P.A., Pohlhaus P.D., Luft J.C., Madden V.J., Napier M.E., DeSimone J.M. (2008). The effect of particle design on cellular internalization pathways. Proc. Natl. Acad. Sci. USA.

[20] Oh N., Park J.H. (2014). Endocytosis and exocytosis of nanoparticles in mammalian cells. Int. J. Nanomed..

[21] Murphy F.A., Schinwald A., Poland C.A., Donaldson K. (2012). The mechanism of pleural inflammation by long carbon nanotubes: Interaction of long fibres with macrophages stimulates them to amplify pro-inflammatory responses in mesothelial cells. Part. Fibre Toxicol..

[22] Landsiedel R., Sauer U.G., Ma-Hock L., Schneckenburger J., Wiemann M. (2014). Pulmonary toxicity of nanomaterials—A critical comparison of published *in vitro* assays and *in vivo* inhalation or instillation studies. Nanomedicine.

[23] Comfort K.K., Braydich-Stolle L.K., Maurer E.I., Hussain S.M. (2014). Less is more: Long-term *in vitro* exposure to low levels of silver nanoparticles provides new insights for nanomaterial evaluation. ACS Nano.

[24] Nel A.E., Madler L., Velegol D., Xia T., Hoek E.M., Somasundaran P., Klaessig F., Castranova V., Thompson M. (2009). Understanding biophysicochemical interactions at the nano-bio interface. Nat. Mater..

[25] Wörle-Knirsch J.M., Pulskamp K., Krug H.F. (2006). Oops they did it again! Carbon nanotubes hoax scientists in viability assays. Nano Lett..

[26] Jiang X., Weise S., Hafner M., Rocker C., Zhang F., Parak W.J., Nienhaus G.U. (2010). Quantitative analysis of the protein corona on FePt nanoparticles formed by transferrin binding. J. R. Soc. Interface.

[27] Constantini L., Gilberti R., Knecht D. (2011). The phagocytosis and toxicity of amorphous silica. PLoS ONE.

[28] Park E.J., Park K. (2009). Oxidative stress and pro-inflammatory responses induced by silica nanoparticles *in vivo* and *in vitro*. Toxicol. Lett..

[29] Ye Y., Liu J., Chen M., Sun L., Lan M. (2010). *In vitro* toxicity of silica nanoparticles in myocardial cells. Environ. Toxicol. Pharmacol..

[30] Passagne I., Morille M., Rousset M., Pujalte I., L’Azou B. (2012). Implication of oxidative stress in size-dependent toxicity of silica nanoparticles in kidney cells. Toxicology.

[31] Wang F., Gao F., Lan M., Yuan H., Huang Y., Liu J. (2009). Oxidative stress contributes to silica nanoparticle-induced cytotoxicity in human embryonic kidney cells. Toxicol. Vitr..

[32] Wang F., Jiao C., Liu J., Yuan H., Lan M., Gao F. (2011). Oxidative mechanisms contribute to nanosize silican dioxide-induced developmental neurotoxicity in PC12 cells. Toxicol. Vitr..

[33] Akhtar M.J., Ahamed M., Kumar S., Siddiqui H., Patil G., Ashquin M., Ahmad I. (2010). Nanotoxicity of pure silica mediated through oxidant generation rather than glutathione depletion in human lung epithelial cells. Toxicology.

[34] Foldbjerg R., Wang J., Beer C., Thorsen K., Sutherland D.S., Autrup H. (2013). Biological effects induced by BSA-stabilized silica nanoparticles in mammalian cell lines. Chem. Biol. Interact..

[35] Lin W., Huang Y.W., Zhou X.D., Ma Y. (2006). *In vitro* toxicity of silica nanoparticles in human lung cancer cells. Toxicol. Appl. Pharmacol..

[36] Sun L., Li Y., Liu X., Jin M., Zhang L., Du Z., Guo C., Huang P., Sun Z. (2011). Cytotoxicity and mitochondrial damage caused by silica nanoparticles. Toxicol. Vitr..

[37] Yu Y., Duan J., Yu Y., Li Y., Liu X., Zhou X., Ho K.F., Tian L., Sun Z. (2014). Silica nanoparticles induce autophagy and autophagic cell death in hepg2 cells triggered by reactive oxygen species. J. Hazard. Mater..

[38] Lison D., Thomassen L.C., Rabolli V., Gonzalez L., Napierska D., Seo J.W., Kirsch-Volders M., Hoet P., Kirschhock C.E., Martens J.A. (2008). Nominal and effective dosimetry of silica nanoparticles in cytotoxicity assays. Toxicol. Sci..

[39] Liu S., Xu L., Zhang T., Ren G., Yang Z. (2010). Oxidative stress and apoptosis induced by nanosized titanium dioxide in PC12 cells. Toxicology.

[40] Park E.J., Yi J., Chung K.H., Ryu D.Y., Choi J., Park K. (2008). Oxidative stress and apoptosis induced by titanium dioxide nanoparticles in cultured BEAS-2B cells. Toxicol. Lett..

[41] Saquib Q., Al-Khedhairy A.A., Siddiqui M.A., Abou-Tarboush F.M., Azam A., Musarrat J. (2012). Titanium dioxide nanoparticles induced cytotoxicity, oxidative stress and DNA damage in human amnion epithelial (wish) cells. Toxicol. Vitr..

[42] Shukla R.K., Sharma V., Pandey A.K., Singh S., Sultana S., Dhawan A. (2011). Ros-mediated genotoxicity induced by titanium dioxide nanoparticles in human epidermal cells. Toxicol. Vitr..

[43] Long T.C., Tajuba J., Sama P., Saleh N., Swartz C., Parker J., Hester S., Lowry G.V., Veronesi B. (2007). Nanosize titanium dioxide stimulates reactive oxygen species in brain microglia and damages neurons *in vitro*. Environ. Health Perspect..

[44] Sund J., Palomaki J., Ahonen N., Savolainen K., Alenius H., Puustinen A. (2014). Phagocytosis of nano-sized titanium dioxide triggers changes in protein acetylation. J. Proteom..

[45] Karlsson H., Cronholm P., Gustafsson J., Mo L. (2008). CuO NPs are highly toxic—A comparison between metal oxide NPs and CNTs. Chem. Res. Toxicol..

[46] Yu M., Mo Y., Wan R., Chien S., Zhang X., Zhang Q. (2010). Regulation of plasminogen activator inhibitor-1 expression in endothelial cells with exposure to metal nanoparticles. Toxicol. Lett..

[47] Xia T., Kovochich M., Liong M., Madler L., Gilbert B., Shi H., Yeh J.I., Zink J.I., Nel A.E. (2008). Comparison of the mechanism of toxicity of zinc oxide and cerium oxide nanoparticles based on dissolution and oxidative stress properties. ACS Nano.

[48] Rothe G., Valet G. (1990). Flow cytometric analysis of respiratory burst activity in phagocytes with hydroethidine and 2′,7′-dichlorofluorescin. J. Leukoc. Biol..

[49] Bellavite P. (1988). The superoxide-forming enzymatic system of phagocytes. Free Radic. Biol. Med..

[50] Song W., Zhang J., Guo J., Zhang J., Ding F., Li L., Sun Z. (2010). Role of the dissolved zinc ion and reactive oxygen species in cytotoxicity of ZnO nanoparticles. Toxicol. Lett..

[51] Ahamed M., Akhtar M.J., Raja M., Ahmad I., Siddiqui M.K., AlSalhi M.S., Alrokayan S.A. (2011). ZnO nanorod-induced apoptosis in human alveolar adenocarcinoma cells via p53, survivin and bax/bcl-2 pathways: Role of oxidative stress. Nanomedicine.

[52] Alarifi S., Ali D., Alkahtani S., Verma A., Ahamed M., Ahmed M., Alhadlaq H.A. (2013). Induction of oxidative stress, DNA damage, and apoptosis in a malignant human skin melanoma cell line after exposure to zinc oxide nanoparticles. Int. J. Nanomed..

[53] Wang J., Deng X., Zhang F. (2014). ZnO nanoparticle-induced oxidative stress triggers apoptosis by activating JNK signaling pathway in cultured primary astrocytes. Nanoscale Res. Lett..

[54] Huang C.C., Aronstam R.S., Chen D.R., Huang Y.W. (2010). Oxidative stress, calcium homeostasis, and altered gene expression in human lung epithelial cells exposed to ZnO nanoparticles. Toxicol. Vitr..

[55] Fahmy B., Cormier S.A. (2009). Copper oxide nanoparticles induce oxidative stress and cytotoxicity in airway epithelial cells. Toxicol. Vitr..

[56] Wang Z., von dem Bussche A., Kabadi P.K., Kane A.B., Hurt R.H. (2013). Biological and environmental transformations of copper based nanomaterials. ACS Nano.

[57] Mittal S., Pandey A.K. (2014). Cerium oxide nanoparticles induced toxicity in human lung cells: Role of ros mediated DNA damage and apoptosis. BioMed Res. Int..

[58] Horie M., Nishio K., Kato H., Fujita K., Endoh S., Nakamura A., Miyauchi A., Kinugasa S., Yamamoto K., Niki E. (2011). Cellular responses induced by cerium oxide nanoparticles: Induction of intracellular calcium level and oxidative stress on culture cells. J. Biochem..

[59] Park E.J., Choi J., Park Y.K., Park K. (2008). Oxidative stress induced by cerium oxide nanoparticles in cultured BEAS-2B cells. Toxicology.

[60] Ting S.R., Whitelock J.M., Tomic R., Gunawan C., Teoh W.Y., Amal R., Lord M.S. (2013). Cellular uptake and activity of heparin functionalised cerium oxide nanoparticles in monocytes. Biomaterials.

[61] Karakoti A.S., Monteiro-Riviere N.A., Aggarwal R., Davis J.P., Narayan R.J., Self W.T., McGinnis J., Seal S. (2008). Nanoceria as antioxidant: Synthesis and biomedical applications. JOM.

[62] Herzog E., Byrne H.J., Davoren M., Casey A., Duschl A., Oostingh G.J. (2009). Dispersion medium modulates oxidative stress response of human lung epithelial cells upon exposure to carbon nanomaterial samples. Toxicol. Appl. Pharmacol..

[63] Pulskamp K., Diabate S., Krug H.F. (2007). Carbon nanotubes show no sign of acute toxicity but induce intracellular reactive oxygen species in dependence on contaminants. Toxicol. Lett..

[64] Yang H., Liu C., Yang D., Zhang H., Xi Z. (2009). Comparative study of cytotoxicity, oxidative stress and genotoxicity induced by four typical nanomaterials: The role of particle size, shape and composition. J. Appl. Toxicol..

[65] Chen B., Liu Y., Song W.M., Hayashi Y., Ding X.C., Li W.H. (2011). *In vitro* evaluation of cytotoxicity and oxidative stress induced by multiwalled carbon nanotubes in murine RAW 264.7 macrophages and human A549 lung cells. Biomed. Environ. Sci..

[66] Zhao X., Liu R. (2012). Recent progress and perspectives on the toxicity of carbon nanotubes at organism, organ, cell, and biomacromolecule levels. Environ. Int..

[67] Cheng W.W., Lin Z.Q., Wei B.F., Zeng Q., Han B., Wei C.X., Fan X.J., Hu C.L., Liu L.H., Huang J.H. (2011). Single-walled carbon nanotube induction of rat aortic endothelial cell apoptosis: Reactive oxygen species are involved in the mitochondrial pathway. Int. J. Biochem. Cell. Biol..

[68] Wang J., Sun P., Bao Y., Dou B., Song D., Li Y. (2012). Vitamin e renders protection to PC12 cells against oxidative damage and apoptosis induced by single-walled carbon nanotubes. Toxicol. Vitr..

[69] Piccinno F., Gottschalk F., Seeger S., Nowack B. (2012). Industrial production quantities and uses of ten engineered nanomaterials in europe and the world. J. Nanopart. Res..

[70] Hendren C.O., Mesnard X., Droge J., Wiesner M.R. (2011). Estimating production data for five engineered nanomaterials as a basis for exposure assessment. Environ. Sci. Technol..

[71] Wang Y., Aker W.G., Hwang H.M., Yedjou C.G., Yu H., Tchounwou P.B. (2011). A study of the mechanism of *in vitro* cytotoxicity of metal oxide nanoparticles using catfish primary hepatocytes and human HepG2 cells. Sci. Total Environ..

[72] De Volder M.F., Tawfick S.H., Baughman R.H., Hart A.J. (2013). Carbon nanotubes: Present and future commercial applications. Science.

[73] NCBI Pubmed. http://www.ncbi.nlm.nih.gov/pubmed.

[74] Sciencedirect Sciencedirect. http://www.sciencedirect.com/.

[75] Simko M., Nosske D., Kreyling W.G. (2014). Metrics, dose, and dose concept: The need for a proper dose concept in the risk assessment of nanoparticles. Int. J. Environ. Res. Public Health.

[76] Teeguarden J.G., Hinderliter P.M., Orr G., Thrall B.D., Pounds J.G. (2007). Particokinetics *in vitro*: Dosimetry considerations for *in vitro* nanoparticle toxicity assessments. Toxicol. Sci..

[77] Liu X., Sun J. (2010). Endothelial cells dysfunction induced by silica nanoparticles through oxidative stress via JNK/P53 and NF-kappaB pathways. Biomaterials.

[78] Fernandez D., Garcia-Gomez C., Babin M. (2013). In vitro evaluation of cellular responses induced by ZnO nanoparticles, zinc ions and bulk ZnO in fish cells. Sci. Total Environ..

[79] Buerki-Thurnherr T., Xiao L., Diener L., Arslan O., Hirsch C., Maeder-Althaus X., Grieder K., Wampfler B., Mathur S., Wick P. (2013). In vitro mechanistic study towards a better understanding of zno nanoparticle toxicity. Nanotoxicology.

[80] Seker S., Elcin A.E., Yumak T., Sinag A., Elcin Y.M. (2014). *In vitro* cytotoxicity of hydrothermally synthesized ZnO nanoparticles on human periodontal ligament fibroblast and mouse dermal fibroblast cells. Toxicol. Vitr..

[81] Setyawati M.I., Tay C.Y., Leong D.T. (2013). Effect of zinc oxide nanomaterials-induced oxidative stress on the p53 pathway. Biomaterials.

[82] Sun J., Wang S., Zhao D., Hun F.H., Weng L., Liu H. (2011). Cytotoxicity, permeability, and inflammation of metal oxide nanoparticles in human cardiac microvascular endothelial cells: Cytotoxicity, permeability, and inflammation of metal oxide nanoparticles. Cell Biol. Toxicol..

[83] Sharma V., Anderson D., Dhawan A. (2012). Zinc oxide nanoparticles induce oxidative DNA damage and ros-triggered mitochondria mediated apoptosis in human liver cells (HepG2). Apoptosis.

[84] Song Y., Guan R., Lyu F., Kang T., Wu Y., Chen X. (2014). *In vitro* cytotoxicity of silver nanoparticles and zinc oxide nanoparticles to human epithelial colorectal adenocarcinoma (Caco-2) cells. Mutat. Res..

[85] Akhtar M.J., Ahamed M., Kumar S., Khan M.M., Ahmad J., Alrokayan S.A. (2012). Zinc oxide nanoparticles selectively induce apoptosis in human cancer cells through reactive oxygen species. Int. J. Nanomed..

[86] Muthuraman P., Ramkumar K., Kim D.H. (2014). Analysis of dose-dependent effect of zinc oxide nanoparticles on the oxidative stress and antioxidant enzyme activity in adipocytes. Appl. Biochem. Biotechnol..

[87] Akhtar M.J., Ahamed M. (2012). Protective effect of sulphoraphene against oxidative stress mediated toxicity induced by CuO nps in mouse embryonic fibroblasts BALB 3T3. J. Toxicol. Sci..

[88] Kumbicak U., Cavas T., Cinkilic N., Kumbicak Z., Vatan O., Yilmaz D. (2014). Evaluation of *in vitro* cytotoxicity and genotoxicity of copper-zinc alloy nanoparticles in human lung epithelial cells. Food Chem. Toxicol..

[89] Xu P., Xu J., Liu S., Yang Z. (2012). Nano copper induced apoptosis in podocytes via increasing oxidative stress. J. Hazard. Mater..

[90] Schubert D., Dargusch R., Raitano J., Chan S.W. (2006). Cerium and yttrium oxide nanoparticles are neuroprotective. Biochem. Biophys. Res. Commun..

[91] Celardo I., de Nicola M., Mandoli C., Pedersen J.Z., Traversa E., Ghibelli L. (2011). Ce^3+^ ions determine redox-dependent anti-apoptotic effect of cerium oxide nps. ACS Nano.

[92] Choi S.J., Oh J.M., Choy J.H. (2009). Toxicological effects of inorganic nanoparticles on human lung cancer A549 cells. J. Inorg. Biochem..

[93] Cicchetti R., Divizia M., Valentini F., Argentin G. (2011). Effects of single-wall carbon nanotubes in human cells of the oral cavity: Geno-cytotoxic risk. Toxicol. Vitr..

[94] Guo Y.Y., Zhang J., Zheng Y.F., Yang J., Zhu X.Q. (2011). Cytotoxic and genotoxic effects of multi-wall carbon nanotubes on human umbilical vein endothelial cells *in vitro*. Mutat. Res..

[95] Kaiser J.P., Buerki-Thurnherr T., Wick P. (2012). Influence of single walled carbon nanotubes at subtoxical concentrations on cell adhesion and other cell parameters of human epithelial cells. J. King Saud Univ. Sci..

[96] Pichardo S., Gutierrez-Praena D., Puerto M., Sanchez E., Grilo A., Camean A.M., Jos A. (2012). Oxidative stress responses to carboxylic acid functionalized single wall carbon nanotubes on the human intestinal cell line Caco-2. Toxicol. Vitr..

[97] Song M., Zeng L., Yuan S., Yin J., Wang H., Jiang G. (2013). Study of cytotoxic effects of single-walled carbon nanotubes functionalized with different chemical groups on human MCF7 cells. Chemosphere.

[98] Thurnherr T., Brandenberger C., Fischer K., Diener L., Manser P., Maeder-Althaus X., Kaiser J.P., Krug H.F., Rothen-Rutishauser B., Wick P. (2011). A comparison of acute and long-term effects of industrial multiwalled carbon nanotubes on human lung and immune cells *in vitro*. Toxicol. Lett..

[99] Thurnherr T., Su D.S., Diener L., Weinberg G., Manser P., Pfänder N., Arrigo R., Schuster M.E., Wick P., Krug H.F. (2009). Comprehensive evaluation ofin vitrotoxicity of three large-scale produced carbon nanotubes on human jurkat t cells and a comparison to crocidolite asbestos. Nanotoxicology.

[100] Wang J., Sun P., Bao Y., Liu J., An L. (2011). Cytotoxicity of single-walled carbon nanotubes on PC12 cells. Toxicol. Vitr..

[101] Ye S.F., Wu Y.H., Hou Z.Q., Zhang Q.Q. (2009). Ros and NF-kappaB are involved in upregulation of IL-8 in A549 cells exposed to multi-walled carbon nanotubes. Biochem. Biophys. Res. Commun..

