# Supplementary material for: Pooling and Analysis of Published in Vitro Data: A Proof of Concept Study for the Grouping of Nanoparticles
Source: Int J Mol Sci. 2015 Nov 2;16(11):26211–36. doi: 10.3390/ijms161125954 (PMC4661813; doi:10.3390/ijms161125954)
Supplement: Supplementary file 1 [file ijms-16-25954-s001.pdf]

International Journal of *Molecular Sciences*

MDPI

Supplementary Information

Table S1. Compilation of selected nanoparticles data.

Data Compilation: SiO_2									
**Ref.**	**Size (nm)**	**Origin of NM/Synthesis Method**	**Cells**	**Organism**	**Phagocytes**	**Pos. Control**	**Concentration (µg/mL)**	**Exp. Time (h)**	**Ratio ROS/Control**
[33]	10	Nanostructured & Amorphous Materials	A549 lung adenocarcinoma epithelial cell	human	n	n	200	48	*1.3*
							400	48	*1.4*
	80	Nanostructured & Amorphous Materials	A549 lung adenocarcinoma epithelial cell	human	n	n	200	48	*1.2*
							400	48	*1.4*
[8]	26	Corpuscular Inc.	Hepatocytes liver	rat	y	y	0.384	4	0.7
							1.92	4	0.7
							9.6	4	0.7
							48	4	0.7
							240	4	0.6
							0.384	24	0.7
							1.92	24	0.5
							9.6	24	0.6
							48	24	0.6
							240	24	0.6
	52	Corpuscular Inc.	Hepatocytes liver	rat	y	y	0.384	4	0.8
							1.92	4	1.0
							9.6	4	1.0
							48	4	0.6
							240	4	0.6
							0.384	24	1.0
							1.92	24	0.5
							9.6	24	0.5
							48	24	0.5
							240	24	*0.4*

Table S1. *Cont.*

Data Compilation: SiO_2									
**Ref.**	**Size (nm)**	**Origin of NM/Synthesis Method**	**Cells**	**Organism**	**Phagocytes**	**Pos. Control**	**Concentration (µg/mL)**	**Exp. Time (h)**	**Ratio ROS/Control**
[34]	25	Sigma Aldrich	A549 lung adenocarcinoma epithelial cell	human	n	n	113	24	2.4
							130	24	2.6
							226	24	2.9
							338	24	3.5
[9]	15	Wan Jing New Material Co., Ltd.	HaCaT epidermal keratinocyte	human	n	n	2.5	24	4.7
							5	24	*6.5*
							10	24	*8.0*
	30	Wan Jing New Material Co., Ltd.	HaCaT epidermal keratinocyte	human	n	n	2.5	24	3.7
							5	24	4.0
							10	24	4.6
	100	Wan Jing New Material Co., Ltd.	HaCaT epidermal keratinocyte	human	n	n	2.5	24	3.2
							5	24	3.3
							10	24	3.7
[55]	30	Alfa Aesar	Hep-2 laryngeal epithelial	human	n	n	125	0.5	1.7
[35]	15	Degussa Co.	A549 lung adenocarcinoma epithelial cell	human	n	n	10	48	1.4
							50	48	*1.6*
							100	48	*1.9*
[77]	20	sol-gel synthesizing	HUVEC umbilical vein endothelial	human	n	n	25	3	1.0
							50	3	1.3
							100	3	1.5
							200	3	2.7
							25	24	1.3
							50	24	1.7
							100	24	2.4
							200	24	*3.2*

Table S1. *Cont.*

Data Compilation: SiO_2									
**Ref.**	**Size (nm)**	**Origin of NM/Synthesis Method**	**Cells**	**Organism**	**Phagocytes**	**Pos. Control**	**Concentration (μg/mL)**	**Exp. Time (h)**	**Ratio ROS/Control**
[28]	12	Degussa Co.	RAW264.7 peritoneal macrophage	mouse	y	n	5	24	1.1
							10	24	*1.2*
							20	24	*1.2*
							40	24	*1.4*
[30]	16	Reverse microemulsion process	HK-2 kidney	human	n	y	5	24	1.1
							10	24	1.1
							25	24	*1.1*
							50	24	*1.3*
			LLC-PK1 tubular	porcine	n	y	5	24	1.3
							10	24	1.4
							25	24	1.6
							50	24	*2.1*
	85	Reverse microemulsion process	HK-2 kidney	human	n	y	5	24	1.0
							10	24	1.1
							25	24	1.1
							50	24	*1.3*
			LLC-PK1 tubular	porcine	n	y	5	24	1.1
							25	24	1.1
							10	24	1.2
							50	24	1.2
[36]	43	Stöber method	HepG2 hepatocellular carcinoma	human	n	n	25	3	1.5
							50	3	2.3
							100	3	2.7
							200	3	2.0
							25	24	1.2

Table S1. *Cont.*

Data Compilation: SiO_2									
**Ref.**	**Size (nm)**	**Origin of NM/Synthesis Method**	**Cells**	**Organism**	**Phagocytes**	**Pos. Control**	**Concentration (μg/mL)**	**Exp. Time (h)**	**Ratio ROS/Control**
							50	24	1.2
							100	24	*1.7*
							200	24	*1.0*
[37]	62	Stöber method	HepG2 hepatocellular carcinoma	human	n	n	25	24	1.7
							50	24	*2.1*
							75	24	*2.8*
							100	24	*3.1*
[32]	20	East China University of Science and Technology	PC12 pheochromocytoma	rat	n	n	25	24	1.5
							50	24	1.9
							100	24	*2.2*
	50	East China University of Science and Technology	PC12 pheochromocytoma	rat	n	n	25	24	1.2
							50	24	1.7
							100	24	*2.0*
[64]	20	Runhe Co., Ltd.	primary embryo fibroblasts	mouse	n	n	5	24	1.2
							10	24	1.5
							20	24	1.5
							50	24	2.2
							100	24	2.6
[29]	21	Sol-gel method. East China University of Science and Technology	H9c2(2-1) embryonic ventricular myocardial	rat	n	n	100	24	1.6
							300	24	*3.1*
			L-02 hepatic	human	n	n	200	24	1.1
							400	24	*1.5*
							600	24	*3.7*
	49	Sol-gel method. East China University of Science and Technology	H9c2(2-1) embryonic ventricular myocardial	rat	n	n	100	24	1.2
							300	24	1.8

Table S1. *Cont.*

**Data Compilation: SiO_2**									
**Ref.**	**Size (nm)**	**Origin of NM/Synthesis Method**	**Cells**	**Organism**	**Phagocytes**	**Pos. Control**	**Concentration (μg/mL)**	**Exp. Time (h)**	**Ratio ROS/Control**
[10]	19	Jilin University	HepG2 hepatocellular carcinoma	human	n	y	100	24	*1.3*
	43	Jilin University	HepG2 hepatocellular carcinoma	human	n	y	100	24	*1.1*
	68	Jilin University	HepG2 hepatocellular carcinoma	human	n	y	100	24	1.1
[31]	20	East China University of Science and Technology	HEK293 embryonic kidney	human	n	n	25	24	*1.3*
							50	24	*1.8*
							100	24	*2.3*
	50	East China University of Science and Technology	HEK293 embryonic kidney	human	n	n	25	24	1.1
							50	24	*1.4*
							100	24	*2.2*
**Data Compilation: TiO_2**									
**Ref.**	**Size (nm)**	**Origin of NM/Synthesis Method**	**Cells**	**Organism**	**Phagocytes**	**Pos. Control**	**Concentration (μg/mL)**	**Exp. Time (h)**	**Ratio ROS/Control**
[2]	22	Degussa	Hepatocytes liver	rat	y	y	0.384	4	0.9
							1.92	4	0.7
							9.6	4	0.6
							48	4	0.6
							240	4	0.6
							0.384	24	0.4
							1.92	24	0.3
							9.6	24	0.3
							48	24	0.2
							240	24	0.3
[39]	21		PC12 pheochromocytoma	rat	n	n	10	24	*1.2*
							50	24	*1.5*
							100	24	*1.8*
							30	0.1	1.3

Table S1. *Cont.*

Data Compilation: TiO_2									
**Ref.**	**Size (nm)**	**Origin of NM/Synthesis Method**	**Cells**	**Organism**	**Phagocytes**	**Pos. Control**	**Concentration (µg/mL)**	**Exp. Time (h)**	**Ratio ROS/Control**
							15	0.1	1.3
							60	0.1	1.7
							120	0.1	2.5
[40]	21	Degussa Korea	BEAS-2b bronchial epithelial	human	n	n	5	24	1.2
							10	24	*1.3*
							20	24	*1.5*
							40	24	*1.7*
[41]	31	Sonomechanical synthesis	WISH amnion epithelial	human	n	y	0.625	24	1
							1.25	24	*1*
							2.5	24	*1*
							5	24	*1.3*
							10	24	*1.9*
[42]	50	Sigma Chemical Co. Ltd.	A431 epidermal	human	n	n	0.008	6	1.2
							0.08	6	1.3
							0.8	6	1.5
							8	6	1.7
							80	6	1.8
[44]	35	NanoAmor. Nanostructured and Amorphous Materials Inc.	THP-1 monocytic leukemia	human	y	y	3	3	0.4
							30	3	3.5
							300	3	3.4
[47]	11	Flame spray pyrolysis (FSP)	BEAS-2b bronchial epithelial	human	n	n	10	1	1.1
							10	3	0.9
							10	6	0.7
							10	16	1.1
			RAW 264.7 macrophage	mouse	y	n	10	1	2.5

Table S1. *Cont.*

Data Compilation: TiO_2									
**Ref.**	**Size (nm)**	**Origin of NM/Synthesis Method**	**Cells**	**Organism**	**Phagocytes**	**Pos. Control**	**Concentration (µg/mL)**	**Exp. Time (h)**	**Ratio ROS/Control**
							10	3	1.8
							10	6	1.3
							10	16	1.2
[46]	28	INABTA and Co.	MPMVEC pulmonary microvascular endothelial	mouse	n	n	0.625	12	1
							1.25	12	1
							2.5	12	1
[11]	12	Hangzhou Wanjingxin Material Co., Ltd.	Ana-1 macrophage	mouse	y	n	12.5	24	1.3
							25	24	1.8
							50	24	2.5
							100	24	*3.3*
							200	24	*4.2*
							400	24	*6.4*
							600	24	*7.5*
	21	Hangzhou Wanjingxin Material Co., Ltd.	Ana-1 macrophage	mouse	y	n	12.5	24	1.4
							25	24	*2.1*
							50	24	*3.7*
							100	24	*5.7*
							200	24	*6.7*
							400	24	*9*
							600	24	*12.2*
	98	Hangzhou Wanjingxin Material Co., Ltd.	Ana-1 macrophage	mouse	y	n	12.5	24	1.3
							25	24	1.9
							50	24	2.7
							100	24	*3.5*
							200	24	*4*
							400	24	*5.9*

Table S1. *Cont.*

Ref.	Size (nm)	Origin of NM/Synthesis Method	Cells	Organism	Phagocytes	Pos. Control	Concentration (µg/mL)	Exp. Time (h)	Ratio ROS/Control
**Data Compilation: TiO_2**									
							600	24	*6.8*
	148	Hangzhou Wanjingxin Material Co., Ltd.	Ana-1 macrophage	mouse	y	n	12.5	24	1.4
							25	24	1.6
							50	24	2.3
							100	24	2.5
							200	24	*3.1*
							400	24	*3.8*
							600	24	*4.7*
[45]	63	Sigma-Aldrich	A549 lung adenocarcinoma epithelial cell	human	n	n	40	4	1.4
							80	4	1.6

Ref.	Size (nm)	Origin of NM/Synthesis Method	Cells	Organism	Phagocytes	Pos. Control	Concentration (µg/mL)	Exp. Time (h)	Ratio ROS/Control
**Data Compilation: ZnO**									
[51]	50	Sol-gel synthesis	A549 lung adenocarcinoma epithelial cell	human	n	n	5	24	1.1
							10	24	*1.3*
							25	24	*1.7*
							50	24	*2.0*
							100	24	*2.5*
							5	48	1.1
							10	48	*1.6*
							25	48	*1.8*
							50	48	*2.3*
							100	48	*2.9*
[52]	17	Sigma Aldrich	A375 malignant melanoma skin	human	n	n	5	24	1.1
							10	24	*1.2*
							20	24	*1.6*

Table S1. *Cont.*

Data Compilation: ZnO									
**Ref.**	**Size (nm)**	**Origin of NM/Synthesis Method**	**Cells**	**Organism**	**Phagocytes**	**Pos. Control**	**Concentration (μg/mL)**	**Exp. Time (h)**	**Ratio ROS/Control**
							5	48	1.2
							10	48	*1.4*
							20	48	*1.8*
[78]	58	Sigma Aldrich	RTG-2 gonad tissue	fish	n	y	3.12	24	1.2
							6.25	24	*1.3*
							12.5	24	*1.4*
							25	24	*1.5*
							3.12	48	1.1
							6.25	48	*1.2*
							12.5	48	*1.4*
							25	48	*1.5*
			RTH-149 epithelial	fish	n	y	3.12	24	1.1
							6.25	24	*1.3*
							12.5	24	*1.4*
							25	24	*1.4*
							3.12	48	1.2
							6.25	48	1.4
							12.5	48	1.5
							25	48	*1.6*
			RTL-W1 liver	fish	n	y	3.12	24	1.4
							6.25	24	1.5
							12.5	24	1.5
							25	24	*1.6*
							3.12	48	1.4
							6.25	48	1.5
							12.5	48	1.6

Table S1. *Cont.*

Data Compilation: ZnO									
**Ref.**	**Size (nm)**	**Origin of NM/Synthesis Method**	**Cells**	**Organism**	**Phagocytes**	**Pos. Control**	**Concentration (μg/mL)**	**Exp. Time (h)**	**Ratio ROS/Control**
							25	48	*1.6*
[54]	20	Nanostructured and Amorphous Materials	BEAS-2b bronchial epithelial	human	n	n	5	6	1.4
							6	6	2.0
							7	6	*2.2*
							8	6	*3.2*
							9	6	*3.8*
							10	6	*4.0*
							5	24	1.3
							6	24	1.4
							7	24	*1.9*
							8	24	*4.1*
							9	24	*7.4*
							10	24	*11.6*
[79]	10	IBU-tec advanced materials AG	Jurkat A3 leukemic T cell	human	n	y	1	2	1.0
							5	2	1.0
							10	2	1.0
							50	2	1.7
							100	2	2.1
[80]	155	Hydrothermal method	hPDLF peridontal ligament fibroblast tissue	human	n	y	0.1	0.5	1.0
							1	0.5	1.1
							10	0.5	1.1
							50	0.5	1.3
							100	0.5	1.3
			mDF dermal fibroblast	mouse	n	y	0.1	0.5	1.0
							1	0.5	1.1
							10	0.5	1.2

Table S1. *Cont.*

Data Compilation: ZnO									
**Ref.**	**Size (nm)**	**Origin of NM/Synthesis Method**	**Cells**	**Organism**	**Phagocytes**	**Pos. Control**	**Concentration (μg/mL)**	**Exp. Time (h)**	**Ratio ROS/Control**
							50	0.5	1.3
							100	0.5	1.3
[81]	31	Sigma Aldrich	BJ shp53 (knockdown)	human	n	n	0.5	5	1.0
							1	5	1.1
							2	5	1.2
							4	5	1.4
							8	5	1.6
							12	5	1.8
							16	5	2.1
							20	5	2.5
			BJ WT neonatal foreskin fibroblast	human	n	n	0.5	5	1.0
							1	5	1.0
							2	5	1.0
							4	5	1.0
							8	5	1.3
							12	5	1.7
							16	5	2.2
							20	5	3.0
[50]	19	Shenzhen Nanuo Nanomaterials Corp.	Ana-1 macrophage	mouse	y	n	5	24	*0.5*
							10	24	*0.8*
							20	24	*1.5*
							40	24	*2.3*
							80	24	*3.2*
							100	24	*3.8*

Table S1. *Cont.*

Data Compilation: ZnO									
**Ref.**	**Size (nm)**	**Origin of NM/Synthesis Method**	**Cells**	**Organism**	**Phagocytes**	**Pos. Control**	**Concentration (μg/mL)**	**Exp. Time (h)**	**Ratio ROS/Control**
	71	Beijing Nachen Technology Co., Ltd.	Ana-1 macrophage	mouse	y	n	5	24	0.6
							10	24	*0.7*
							20	24	*0.9*
							40	24	*1.2*
							80	24	*2.2*
							100	24	*2.7*
	108	Beijing Nachen Technology Co., Ltd.	Ana-1 macrophage	mouse	y	n	5	24	0.5
							10	24	*0.6*
							20	24	*0.9*
							40	24	*1.4*
							80	24	*2.4*
							100	24	*2.8*
	342	Hangzhou Wanjingxin Material Co., Ltd.	Ana-1 macrophage	mouse	y	n	5	24	0.4
							10	24	0.6
							20	24	*1.0*
							40	24	*1.7*
							80	24	*2.6*
							100	24	*3.0*
[82]	45	Sigma-Aldrich	HCMEC cardiac microvascular endothelial	human	n	n	0.001	12	0.9
							0.01	12	1.2
							0.1	12	1.0
							1	12	1.2
							5	12	1.3
							10	12	*1.6*
							20	12	*3.3*

Table S1. *Cont.*

Data Compilation: ZnO									
**Ref.**	**Size (nm)**	**Origin of NM/Synthesis Method**	**Cells**	**Organism**	**Phagocytes**	**Pos. Control**	**Concentration (μg/mL)**	**Exp. Time (h)**	**Ratio ROS/Control**
							50	12	*4.3*
							100	12	*5.2*
							0.001	24	1.0
							0.01	24	1.2
							0.1	24	1.0
							1	24	1.0
							5	24	*3.4*
							10	24	*4.3*
							20	24	*6.4*
							50	24	*7.3*
							100	24	*7.1*
[53]	45	HAT Nano Company	astrocytes	rat	n	n	4	6	1.2
							8	6	*1.4*
							12	6	*2.1*
							4	12	0.9
							8	12	*1.0*
							12	12	*2.2*
							4	24	1.0
							8	24	*1.0*
							12	24	*1.1*
[47]	13	Flame spray pyrolysis (FSP)	BEAS-2b bronchial epithelial	human	n	n	10	1	0.9
							10	3	0.7
							10	6	0.7
							10	16	0.0
			RAW 264.7	mouse	y	n	10	1	6.7

Table S1. *Cont.*

Data Compilation: ZnO									
**Ref.**	**Size (nm)**	**Origin of NM/Synthesis Method**	**Cells**	**Organism**	**Phagocytes**	**Pos. Control**	**Concentration (μg/mL)**	**Exp. Time (h)**	**Ratio ROS/Control**
							10	3	6.5
							10	6	4.7
							10	16	2.6
[64]	20	Nanuo Co., Ltd.	Primary embryo fibroblasts	mouse	n	n	5	24	1.3
							10	24	*2.3*
							20	24	*3.0*
							50	24	*4.9*
							100	24	*4.9*
[83]	30	Sigma Chemical Co., Ltd.	HepG2 hepatocellular carcinoma	human	n	n	8	6	1.0
							14	6	1.2
							20	6	1.3
[84]	90	Hangzhou Wan Jing New Ltd.	Caco-2 epithelial colorectal adenocarcinoma	human	n	n	10	24	*2.3*
							25	24	*2.3*
							50	24	*2.3*
							100	24	*2.3*
							200	24	*2.3*
[85]	22	Coprecipitation synthesis	HepG2 hepatocellular carcinoma	human	n	n	15	24	*1.5*
[45]	71	Sigma-Aldrich	A549 lung adenocarcinoma epithelial cell	human	n	n	40	4	1.1
							80	4	1.3
[86]	35	Chemical pyrolysis	3T3-L1 preadipocytes	mouse	n	n	10	48	1.2
							20	48	*1.3*
							30	48	*1.5*
							40	48	*1.6*
							50	48	*1.8*
							60	48	*1.9*

Table S1. *Cont.*

Data Compilation: ZnO									
**Ref.**	**Size (nm)**	**Origin of NM/Synthesis Method**	**Cells**	**Organism**	**Phagocytes**	**Pos. Control**	**Concentration (μg/mL)**	**Exp. Time (h)**	**Ratio ROS/Control**
							70	48	*2.1*
							80	48	*2.2*
**Data Compilation: CuO**									
**Ref.**	**Size (nm)**	**Origin of NM/Synthesis Method**	**Cells**	**Organism**	**Phagocytes**	**Pos. Control**	**Concentration (μg/mL)**	**Exp. Time (h)**	**Ratio ROS/Control**
[87]	26	Aquaeous precipitation	BALB3T3 embryonic fibroblasts	mouse	n	n	5	48	1.3
							10	48	*1.6*
							15	48	*1.8*
[55]	30	Alfa Aesar	Hep-2 laryngeal epithelial	human	n	n	125	0.5	1.3
[45]	42	Sigma-Aldrich	A549 lung adenocarcinoma epithelial cell	human	n	n	40	4	1.4
							80	4	2.1
[88]	180	Sigma Aldrich	BEAS-2b bronchial epithelial	human	n	n	0.1	3	1.2
							0.2	3	1.3
							6.4	3	1.4
							3.2	3	1.4
							0.4	3	1.5
							0.8	3	1.6
							1.6	3	1.6
[82]	46	Sigma-Aldrich	HCMEC cardiac microvascular endothelial	human	n	n	0.001	12	1
							0.01	12	1.2
							0.1	12	1.2
							1	12	1.3
							5	12	*1.5*
							10	12	*1.8*
							20	12	*2.8*

Table S1. *Cont.*

Data Compilation: CuO									
**Ref.**	**Size (nm)**	**Origin of NM/Synthesis Method**	**Cells**	**Organism**	**Phagocytes**	**Pos. Control**	**Concentration (μg/mL)**	**Exp. Time (h)**	**Ratio ROS/Control**
							50	12	*4.6*
							100	12	*4.8*
							0.01	24	0.9
							0.1	24	1
							0.001	24	1.2
							1	24	1.2
							5	24	*2.1*
							10	24	*3.1*
							20	24	*6.1*
							50	24	*7.3*
							100	24	*7*
[56]	50	Sigma Aldrich	J774.A1 macrophage	mouse	y	n	2.5	0.5	1
							5	0.5	1.8
							10	0.5	3.6
[89]	15	University of Hertfordshire	podocytes	mouse	n	n	1	0.5	1.1
							10	0.5	1.3
							30	0.5	1.5
[46]	42	Sigma-Aldrich	MPMVEC pulmonary microvascular endothelial	mouse	n	n	0.625	12	1.4
							1.25	12	1.8
							2.5	12	2.1

Table S1. *Cont.*

Data Compilation: CeO_2									
**Ref.**	**Size (nm)**	**Origin of NM/Synthesis Method**	**Cells**	**Organism**	**Phagocytes**	**Pos. Control**	**Concentration (µg/mL)**	**Exp. Time (h)**	**Ratio ROS/Control**
[58]	14	C.I. Kasei Company Ltd./physical vapour synthesis	HaCaT epidermal keratinocyte	human	n	n	100	2	0.7
							100	6	0.6
							100	12	0.6
							100	24	0.8
							200	2	0.8
							200	6	0.6
							200	12	0.9
							200	24	1.5
			A549 lung adenocarcinoma epithelial cell	human	n	n	100	2	0.8
							100	6	0.6
							100	12	0.7
							100	24	0.5
							200	2	0.8
							200	6	*1.0*
							200	12	0.9
							200	24	*0.9*
	400	Junsei Chemical Company Ltd.	HaCaT epidermal keratinocyte	human	n	n	100	2	0.9
							100	6	0.8
							100	12	0.8
							100	24	0.9
							200	2	1.1
							200	6	0.9
							200	12	1.4
							200	24	*1.3*

Table S1. *Cont.*

Data Compilation: CeO_2									
**Ref.**	**Size (nm)**	**Origin of NM/Synthesis Method**	**Cells**	**Organism**	**Phagocytes**	**Pos. Control**	**Concentration (µg/mL)**	**Exp. Time (h)**	**Ratio ROS/Control**
			A549 lung adenocarcinoma epithelial cell	human	n	n	100	2	1.2
							100	6	0.8
							100	12	0.9
							100	24	0.9
							200	2	0.7
							200	6	0.8
							200	12	1.0
							200	24	1.1
[59]	30	Supercritical synthesis	BEAS-2b bronchial epithelial	human	n	n	5	24	1.1
							10	24	1.2
							20	24	*1.3*
							40	24	*1.4*
[90]	12	Mixing of Ce(NO3)3Æ6H2O with HMT	HT22 hippocampal nerve cell line	mouse	n	y	20	0.2	0.6
							20	0.5	0.7
							20	1	0.5
[7]	7	Flame spray pyrolysis	U937 leukemic monocyte lymphoma cell line	human	y	n	50	24	0.8
							50	48	0.4
							50	72	0.9
			U937 (PMA activated) leukemic monocyte lymphoma cell line	human	y	n	50	24	0.9
							50	48	0.6
							50	72	0.8
	14	Flame spray pyrolysis	U937 leukemic monocyte lymphoma cell line	human	y	n	50	24	0.6
							50	48	0.4
							50	72	0.8

Table S1. *Cont.*

Data Compilation: CeO_2									
**Ref.**	**Size (nm)**	**Origin of NM/Synthesis Method**	**Cells**	**Organism**	**Phagocytes**	**Pos. Control**	**Concentration (μg/mL)**	**Exp. Time (h)**	**Ratio ROS/Control**
			U937 (PMA activated) leukemic monocyte lymphoma cell line	human	y	n	50	24	0.8
							50	48	0.4
							50	72	0.6
	94	Flame spray pyrolysis	U937 leukemic monocyte lymphoma cell line	human	y	n	50	24	0.6
							50	48	0.6
							50	72	0.7
			U937 (PMA activated) leukemic monocyte lymphoma cell line	human	y	n	50	24	0.9
							50	48	0.7
							50	72	0.7
[90]	20	Sigma Chemical Co., Ltd.	A549 lung adenocarcinoma epithelial cell	human	n	y	1	3	1.0
							10	3	1.4
							25	3	1.7
							50	3	2.0
							100	3	2.6
							1	6	1.1
							10	6	2.1
							25	6	2.4
							50	6	2.7
							100	6	2.9
							1	24	0.9
							10	24	0.9
							25	24	1.2
							50	24	*1.2*
							100	24	*1.1*

Table S1. *Cont.*

Data Compilation: CeO_2									
**Ref.**	**Size (nm)**	**Origin of NM/Synthesis Method**	**Cells**	**Organism**	**Phagocytes**	**Pos. Control**	**Concentration (μg/mL)**	**Exp. Time (h)**	**Ratio ROS/Control**
[47]	8	Flame spray pyrolysis (FSP)	BEAS-2b bronchial epithelial	human	n	n	10	1	1.1
							10	3	1.1
							10	6	1.0
							10	16	0.9
			RAW 264.7 macrophages	mouse	y	n	10	1	2.1
							10	3	2.1
							10	6	1.8
							10	16	1.2
[60]	7	Flame spray pyrolysis	U937 leukemic monocyte lymphoma cell line	human	y	n	50	24	0.9
							200	24	0.8
							50	48	0.8
							200	48	0.6
							50	72	0.8
							200	72	0.7
			U937 (PMA activated) leukemic monocyte lymphoma cell line	human	y	n	50	24	0.9
							200	24	0.8
							50	48	0.7
							200	48	0.6
							50	72	0.9
							200	72	0.7
[91]	10	Wet-chemical synthesis at room temperature	Jurkat tumour T lymphocytes	human	n	y	200	1	0.9
							200	3	0.9
							200	24	0.8
							200	48	0.6
							200	72	0.5

Table S1. *Cont.*

Ref.	Size (nm)	Origin of NM/Synthesis Method	Cells	Organism	Phagocytes	Pos. Control	Concentration (μg/mL)	Exp. Time (h)	Ratio ROS/Control
**Data Compilation: CeO_2**									
			U937 tumour monocytes	human	y	y	5	1	1.0
							20	1	0.9
							100	1	0.9
							200	1	0.8
							200	3	0.8
							5	6	0.9
							20	6	0.8
							100	6	0.8
							200	6	0.7
							200	6	0.8
							200	24	0.6
							200	48	0.4
							200	72	0.4

Ref.	Size (nm)	Origin of NM/Synthesis Method	Cells	Organism	Phagocytes	Pos. Control	Concentration (μg/mL)	Exp. Time (h)	Ratio ROS/Control
**Data Compilation: CNT**									
[91]	9.5 nm × < 1 μm	Nanocyl	A549 lung adenocarcinoma epithelial cell	human	n	y	25	0.5	5.4
							25	1	17.0
							25	1.5	18.3
							25	2	10.4
							25	3	6.9
							25	4	6.4
							2.5	1	2.7
							10	1	6.2
							25	1	14.9

Table S1. *Cont.*

Ref.	Size (nm)	Origin of NM/Synthesis Method	Cells	Organism	Phagocytes	Pos. Control	Concentration (µg/mL)	Exp. Time (h)	Ratio ROS/Control
**Data Compilation: CNT**									
							100	1	18.9
			RAW 264.7 macrophages	murine	y	y	25	0.5	2.2
							25	1	10.3
							25	1.5	9.4
							25	2	7.2
							25	3	4.3
							25	4	3.1
							2.5	1	4.5
							10	1	12.2
							25	1	28.7
							100	1	35.7
[67]	0.8–1.2 nm × Z µm	Sigma Aldrich	RAEC rat aortic endothelial cells	rat	n	n	12.5	72	1.0
							12.5	96	0.9
							25	72	1.1
							25	96	1.2
							50	72	*1.2*
							50	96	*1.2*
							100	72	*1.4*
							100	96	*2.1*
							200	72	*1.5*
							200	96	3.3
[92]	1.2–1.5 nm × 2.5 µm	Sigma	A549 lung adenocarcinoma epithelial cell	human	n	n	125	72	*4.9*
							250	72	*13.8*
							500	72	*19.2*

Table S1. *Cont.*

Data Compilation: CNT									
**Ref.**	**Size (nm)**	**Origin of NM/Synthesis Method**	**Cells**	**Organism**	**Phagocytes**	**Pos. Control**	**Concentration (µg/mL)**	**Exp. Time (h)**	**Ratio ROS/Control**
[93]	1.6 nm × 0.8 µm	CHeapTubes	HGF gingival fibroblast	human	n	n	50	24	2.3
							75	24	3.1
							100	24	4.3
							125	24	5.1
							150	24	5.7
[94]	30 nm × < 1 µm	Lawrence Berkeley National Laboratory; chemical vapour deposition method	HUVECs umbilical vein endothelial cells	human	n	y	0.5	2	1.1
							5	2	1.2
							20	2	1.9
[95]	50 nm × 0.5–3 µm	Yangtse Nanotechnology. arc-discharged evaporation	A549 lung adenocarcinoma epithelial cell	human	n	y	15	1	2.6
							15	1	2.7
							30	1	4.5
							30	1	5.6
[45]	110–170 nm × 5–9 µm	Sigma-Aldrich	A549 lung adenocarcinoma epithelial cell	human	n	n	40	4	0.8
							80	4	0.9
[96]	15 nm × 0.4–1.2 µm	SW-COOH; Carboxyl functionalized. Sigma Aldrich	Caco-2 epithelial colorectal adenocarcinoma	human	n	n	5	24	0.9
							10	24	1.0
							50	24	1.1
							100	24	1.6
							500	24	3.1
							1000	24	5.1
[62]	10–20 nm × 10–13 µm	Nanostructured & Amorphous Materials	NR8383 alveolar macrophage	rat	y	n	5	24	*1.8*
							10	24	*2.2*
							50	24	*2.5*
							100	24	*3.7*

Table S1. *Cont.*

Data Compilation: CNT									
**Ref.**	**Size (nm)**	**Origin of NM/Synthesis Method**	**Cells**	**Organism**	**Phagocytes**	**Pos. Control**	**Concentration (μg/mL)**	**Exp. Time (h)**	**Ratio ROS/Control**
	1–2 nm × 5–15 μm	Nanostructured & Amorphous Materials	NR8383 alveolar macrophage	rat	y	n	5	24	*1.8*
							10	24	*3.6*
							50	24	*4*
							100	24	*2.5*
	30–50 nm × 10–20 μm	Nanostructured & Amorphous Materials	NR8383 alveolar macrophage	rat	y	n	5	24	*1.3*
							10	24	*1.6*
							50	24	*1.9*
							100	24	*3.2*
[97]	4–5 nm × 0.5–0.6 μm	SW-PEG: PEG functionalized. Sigma Aldrich	MCF-7 breast cancer	human	n	y	5	1	0.9
							10	1	0.8
							20	1	0.8
							40	1	1.0
							80	1	0.7
	4–5 nm × 0.5–1.5 μm	SW-COOH; Carboxyl functionalized. Sigma Aldrich	MCF-7 breast cancer	human	n	y	5	1	0.9
							10	1	1.1
							20	1	0.9
							40	1	0.7
							80	1	0.5
	4–6 nm × 0.7–1 μm	SW-NH3: Amino functionalized. Sigma Aldrich	MCF-7 breast cancer	human	n	y	5	1	1.0
							10	1	0.9
							20	1	1.0
							40	1	1.1
							80	1	1.1

Table S1. *Cont.*

Data Compilation: CNT									
**Ref.**	**Size (nm)**	**Origin of NM/Synthesis Method**	**Cells**	**Organism**	**Phagocytes**	**Pos. Control**	**Concentration (µg/mL)**	**Exp. Time (h)**	**Ratio ROS/Control**
[98]	8–14 nm × 2–4 µm	Bayer Technologies Service	A549 lung adenocarcinoma epithelial cell	human	n	y	3.2	2	17.9
							6.25	2	33.8
							12.5	2	55.6
							25	2	70.5
[69]	1–2 nm × 20 µm	Beijing Nachen Technology & Development Co., Ltd.	PC12 adrenal gland	rat	n	n	50	24	1.6
							50	48	2.1
[99]	10–18 nm × 2–6 µm	Cheap Tubes	Jurkat T cells	human	n	y	7.5	2	2.5
							15	2	3.7
							30	2	4.7
	6–10 nm × 1–2 µm	Nanocyl SA	Jurkat T cells	human	n	y	7.5	2	2.7
							15	2	4.4
							30	2	5.2
	8–14 nm × 2–4 µm	Bayer Technologies Service	Jurkat T cells	human	n	y	7.5	2	5.4
							15	2	7.9
							30	2	10.2
[100]	1–2 nm × 20 µm	Beijing Nachen Technology & Development Co., Ltd.	PC12 adrenal gland	rat	n	n	5	24	*1.4*
							5	48	*1.5*
							50	24	*1.6*
							50	48	*2.1*
							100	24	*2.5*
							100	48	*2.7*
							200	24	*5.0*
							200	48	*5.3*

Table S1. *Cont.*

Data Compilation: CNT									
**Ref.**	**Size (nm)**	**Origin of NM/Synthesis Method**	**Cells**	**Organism**	**Phagocytes**	**Pos. Control**	**Concentration (μg/mL)**	**Exp. Time (h)**	**Ratio ROS/Control**
[64]	8 nm × 5 μm	COCC. Chinese Academy of Science	Primary embryo fibroblasts	mouse	n	n	5	24	1.1
							10	24	2.3
							20	24	*2.6*
							50	24	*4.0*
							100	24	*4.2*
[101]	20–40 nm × 5–30 μm	Nanotech Port	A549 lung adenocarcinoma epithelial cell	human	n	n	50	24	*1.5*
							100	24	*1.9*
							150	24	*2.1*
[62]	0.8–1.2 nm × 800 nm	HiPco. Carbon Nanotechnologies	A549 lung adenocarcinoma epithelial cell	human	n	y	50	1	1.0
	1.2–1.5 nm × 2–5 μm	ArcD. arc discharged. Sigma Aldrich	NHBE normal human primary bronchial epithelial	human	n	y	50	1	0.8
